# Metabarcoding in two isolated populations of wild roe deer (*Capreolus capreolus)* reveals variation in gastrointestinal nematode community composition between regions and among age classes

**DOI:** 10.1186/s13071-021-05087-5

**Published:** 2021-12-04

**Authors:** Camille Beaumelle, Elizabeth M. Redman, Jill de Rijke, Janneke Wit, Slimania Benabed, François Debias, Jeanne Duhayer, Sylvia Pardonnet, Marie-Thérèse Poirel, Gilles Capron, Stéphane Chabot, Benjamin Rey, Glenn Yannic, John S. Gilleard, Gilles Bourgoin

**Affiliations:** 1 Université Lyon 1, CNRS, UMR 5558, Laboratoire de Biométrie et Biologie Evolutive, Université de Lyon, 69100 Villeurbanne, France; 2 Comparative Biology and Experimental Medicine, Host-Parasites Interactions Program, Faculty of Veterinary Medicine, University of Calgary, Calgary, AB Canada; 3 Université Grenoble Alpes, Université Savoie Mont Blanc, CNRS, LECA, 38000 Grenoble, France; 4 VetAgro Sup, Campus Vétérinaire de Lyon, Université de Lyon, 69280 Marcy l’Etoile, France; 5 Office Français de la Biodiversité, 75008 Paris, France

**Keywords:** Strongyle, Ungulate, Parasitism, Amplicon sequence variants, *Haemonchus contortus*, Wildlife, Diversity index

## Abstract

**Background:**

Gastrointestinal nematodes are ubiquitous for both domestic and wild ungulates and have varying consequences for health and fitness. They exist as complex communities of multiple co-infecting species, and we have a limited understanding of how these communities vary in different hosts, regions and circumstances or of how this affects their impacts.

**Methods:**

We have undertaken ITS2 rDNA nemabiome metabarcoding with next-generation sequencing on populations of nematode larvae isolated from 149 fecal samples of roe deer of different sex and age classes in the two isolated populations of Chizé and Trois Fontaines in France not co-grazing with any domestic ungulate species.

**Results:**

We identified 100 amplified sequence variants (ASVs) that were assigned to 14 gastrointestinal nematode taxa overall at either genus (29%) or species (71%) level. These taxa were dominated by parasites classically found in cervids—e.g. *Ostertagia leptospicularis*, *Spiculopteragia* spp. Higher parasite species diversity was present in the Trois Fontaines population than in the Chizé population including the presence of species more typically seen in domestic livestock (*Haemonchus contortus*, *Bunostomum* sp., *Cooperia punctata*, *Teladorsagia circumcincta*). No differences in parasite species diversity or community composition were seen in the samples collected from three zones of differing habitat quality within the Chizé study area. Young roe deer hosted the highest diversity of gastrointestinal nematodes, with more pronounced effects of age apparent in Trois Fontaines. The effect of host age differed between gastrointestinal nematode species, e.g. there was little effect on *O. leptospicularis* but a large effect on *Trichostrongylus* spp. No effect of host sex was detected in either site.

**Conclusions:**

The presence of some livestock parasite species in the Trois Fontaines roe deer population was unexpected given the isolation of this population away from grazing domestic livestock since decades. Overall, our results illustrate the influence of host traits and the local environment on roe deer nemabiome and demonstrate the power of the nemabiome metabarcoding approach to elucidate the composition of gastrointestinal nematode communities in wildlife.

**Graphical Abstract:**

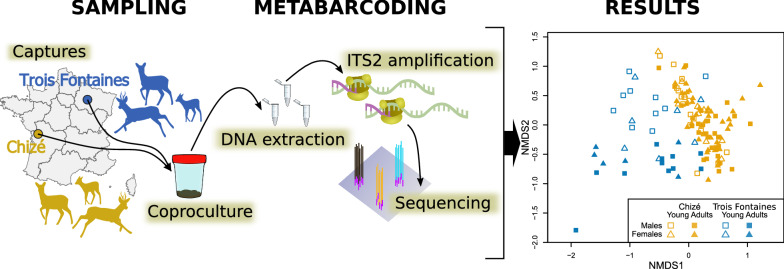

**Supplementary Information:**

The online version contains supplementary material available at 10.1186/s13071-021-05087-5.

## Background

Gastrointestinal nematode parasites of wildlife and livestock have a worldwide distribution. Multiple species usually co-infect a single host, and they have major ecological, economic and animal welfare impacts [1–3]. Gastrointestinal nematodes of wildlife can have negative impacts on the health and individual fitness of hosts [4] as well as on their population dynamics [5], which, in extreme cases, can lead to population extinction [6]. Consequently, understanding host-parasite relationships and determining parasite diversity and abundance are crucial for sustainable wildlife management [7, 8].

The immunological response can help the host to control parasite infestations but it incurs a significant cost to its resources and must be balanced with other energy demanding functions [4, 9], especially when the quality of habitat is poor [10]. Therefore, the physiological and behavioral attributes of individual hosts influence infection level, parasite community composition and their effects [11, 12]. The age, sex and reproductive status of hosts are important factors in this context and determine the parasite community structure, i.e. the abundance and diversity of parasites.

As their immune system is still immature, the highest abundance and diversity of parasites are generally observed in young individuals (e.g. roe deer, *Capreolus capreolus* [13]; Dall’s sheep, *Ovis dalli dalli* [14]; feral sheep, *Ovis aries* [3, 15]). Similarly, because of the deterioration of their immune system, senescent individuals usually host a higher abundance and diversity of parasites than younger adults [16, 17]. Variation in parasite abundance and diversity is also observed between sexes in various species, including ungulates (e.g. roe deer [13], African buffalo, *Syncerus caffer* [18], mouflon, *Ovis gmelini musimon* × *Ovis* sp. [19], feral sheep [3]), and is generally higher in males [20]. The higher susceptibility of males to infection has been ascribed to the immunosuppressive effects of sex steroid hormones in males [21], sexual size dimorphism [22] and also increased likelihood of pathogen exposure [23]. In addition to the influences of life history traits, host populations inhabiting different areas can be infected by various parasites because of the direct effect of environment on parasite communities (e.g. temperature, precipitations) [24] or via the effect of environment on hosts [25].

No relation (e.g. moose, *Alces alces*) [26] or negative relationship (e.g. chamois, Dall’s sheep) [14, 27] on the health and individual fitness of host with increased parasite richness were reported. This might be explained by the nematode community composition with different levels of pathogenicity among parasites, even among closely phylogenetically related species, and may have synergistic or antagonistic interactions [18, 28–30]. Thus, refining the identification of the parasite communities would help to improve our knowledge on host-parasite relationships. However, investigating parasite diversity in wildlife can be challenging [31]. Recently, next-generation sequencing metabarcoding approaches have been developed to investigate gastrointestinal nematode communities [31, 32], although rarely applied to wild ungulates so far [33, 34]. Metabarcoding has a number of advantages including the study of endoparasite communities, without prior assumption of which species are likely to be present. It also allows large numbers of samples to be processed simultaneously and can be highly sensitive to detect rare species [35]. Metabarcoding does not quantify the absolute number of parasites in a sample, but provides information on the proportions of each parasite species.

The aim of this study was to investigate the gastrointestinal nematode communities present in two contrasting natural populations of roe deer (*Capreolus capreolus*), a common and widely distributed ungulate species in Europe. The gastrointestinal nematodes of roe deer have been previously investigated in wild populations [36–39] and have been succinctly described in one of the populations studied here [13]. The two populations of roe deer examined in this study have been spatially isolated in fenced areas for more than 60 years, preventing ungulate movement. Therefore, we expected the diversity of nematodes to be dominated by roe deer-specific parasites (H1). We also expected to observe a higher parasitic diversity in young individuals of a few months of age than in adults (H2) and in males than in females (H3). Because differences in immune responses and differences of body conditions were reported between Chizé and Trois Fontaines [10], we predicted differences of nemabiome with a higher diversity of gastrointestinal nematodes in roe deer living in poor (i.e. Chizé) compared with good environmental conditions (i.e. Trois Fontaines). We predicted similar results at local scale, among the different sectors of Chizé [33], with a higher diversity in the sector with the poorest environmental conditions (H4).

## Materials and methods

### Study areas

**Fig. 1:**
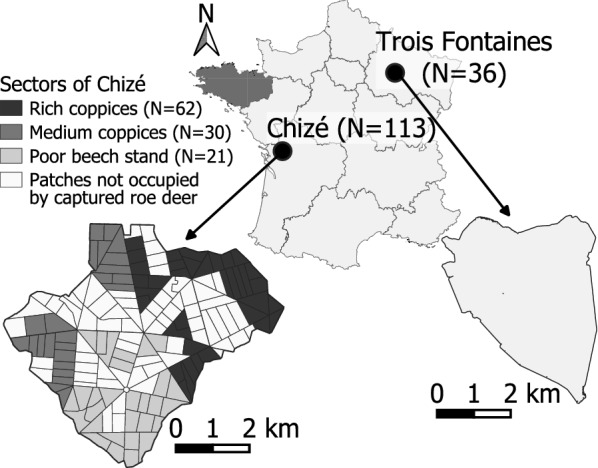
Sampling sites in two regions of France, where roe deer were captured. In Trois Fontaines, habitat is homogeneous with rich coppices, while in Chizé, three sectors with different habitat types and quality are observed (gray color gradient). N corresponds to sampling size within each site

The two study sites, Chizé and Trois Fontaines, are located in western (46°05’ N, 0°25’ W) and northeastern France (48°43’ N, 2°61’ W), respectively. Both sites are fenced forests of 2614 ha in Chizé (since 1978) and 1360 ha in Trois Fontaines (since 1976), preventing roe deer and other ungulates to move into or out of the study sites. No ungulates other than roe deer are present within the two sites, except wild boar. Although some rare hare *Lepus europaeus* inhabit the site of Chizé, there are no lagomorphs in Trois Fontaines (Office Français de la Biodiversité, personal communication). The two sites have different climate, soil and forest attributes. The oceanic climate in Chizé is characterized by mild winters and dry summers in contrast to Trois Fontaines where the continental climate results in rainy warm summers and cold winters. In both sites, forests are dominated by oak (*Quercus* sp.), beech (*Fagus sylvatica*) and hornbeam (*Carpinus betulus*), but with a more heterogeneous spatial distribution of species and coppice abundance and quality in Chizé [40] compared to Trois Fontaines. In Chizé, the variation of coppice type is categorized into three sectors characterized by a rich, medium or poor environment for roe deer (Fig. 1). With its low productive forest, Chizé is still considered a poor habitat for roe deer, in contrast to the highly productive forest of Trois Fontaines [41]. In fact, roe deer in Trois Fontaines have access to high quantity and quality of food in the whole study area, while roe deer in Chizé experience a trade-off between high quantity or quality of forage [42]. Consequently, in Trois Fontaines, the population of roe deer is increasing, whereas the population growth rate is close to 0 in Chizé [10]. Both populations are kept stable by yearly removals (Office Français de la Biodiversité, personal communication).

### Roe deer data

Roe deer of known age are captured annually as part of a long-term monitoring program. Briefly, roe deer were caught using long nets surrounding forest patches and beaters and maintained in wooden boxes until the end of the drive. Details of the capture procedure are available in Gaillard et al. [43]. Fecal samples were collected rectally from immobilized roe deer captured between January and March in 2018 and 2019 under a protocol approved by the Director of Food, Agriculture and Forest (Prefectoral order 2009–14 from Paris) and under authority of the Office Français de la Biodiversité. For each individual, we recorded the sex and age using tooth eruption pattern [43] as well as the approximate location of capture. We also verified that individuals of different age and sex are equally distributed throughout the capture period and spatial localization (data not shown).

### Parasitological analyses and culture

Feces collected from roe deer per rectum were placed in plastic bags and air removed before sealing. In the field, bags were stored in an insulated box to avoid exposure to low temperatures, which could influence survival and development of some parasite species [44]. Samples were then either directly transported or mailed to the parasitology laboratory of VetAgro Sup (Lyon, France) arriving within 24–48 h of collection. On receipt, we set up coprocultures with 5.4 ± 3.7 g (minimum = 1 g, maximum = 23 g) of feces mixed with vermiculite. After an incubation period of 10–12 days at 24 °C with regular mixing and moisture, the larvae present were collected with a Baermann apparatus after 24 h of sedimentation in water. After determining the total number of L3 collected by counting an aliquot, we stored them in 70% ethanol until DNA extraction.

We also counted eggs of gastrointestinal nematodes following a modified McMaster protocol [45], with a solution of ZnSO_4_ (density = 1.36) and a McMaster slide, providing a theoretical sensitivity of 15 eggs per gram (epg) of feces. We also prepared a “control slide” to improve sensitivity of the technique by fully filling a 14-ml tube with the remaining solution and covering it with a coverslip. After centrifugation (5 min at 1200 rpm), the coverslip was transferred on a microscope slide before microscopical observation. We attributed the value 7.5 epg for parasite species detected on the control slide but not on the McMaster slide. Coproscopic data were reported only for samples containing enough L3 collected from coproculture and analyzed by metabarcoding.

### DNA extraction and metabarcoding

DNA extraction was performed using the Qiagen DNeasy® powerSoil kit (Qiagen, Hilden, Germany) following the manufacturer’s instructions with an elution volume of 50–150 µl. ITS2 rDNA metabarcoding was performed using the protocol described by Avramenko et al. [32]. Briefly, the rDNA ITS2 target was PCR-amplified from a 1:10 dilution of genomic DNA template. Negative experimental controls were included consisting of water added to the PCR reaction instead of template DNA. These controls were handled in exactly the same way as the actual samples. All samples (*n* = 149) and negative controls (*n* = 22) were tagged with unique barcode identifiers to allow the pooling (or normalization) into a single 100 ng amplicon library. The final concentration of the pooled library was assessed with the KAPA qPCR Library Quantification Kit (KAPA Biosystems, USA) following the manufacturer's recommended protocol. The prepared pooled library was run on an Illumina MiSeq Desktop Sequencer using a 600-cycle pair-end reagent kit (MiSeq Reagent Kits v3) at a concentration of 15 nM with the addition 25% PhiX Control v3 (Illumina). A standard demultiplexing protocol was employed that generated FASTQ files for all barcoded samples in the pooled library.

### Bioinformatics

All analyses were carried out using R 3.6 (R Core team, 2020). We curated DNA sequence data in two steps (see Additional file 1: Figure S1). First, the sequence reads were passed through a DADA2-based pipeline [46]. Specific details are available at www.nemabiome.ca. Primers were removed with Cutadapt [47] after the removal of ambiguous bases. Next, reads were discarded based on the number of errors (> 2 and > 5 in forward and reverse reads, respectively), length (< 50 bp) and quality (truncG = 2 and phiX genome). Forward and reverse reads were merged only if they overlapped by > 12 bp with low mismatches (i.e. maximum 3 for > 100 bp, maximum 2 for > 50 bp and 1 for < 50 bp reads). Putative chimeras were automatically removed but we verified the effect of this filtering on the data set post-analysis, as these programs are known to produce many false positives [48]. A conservative approach was used for taxonomic assignment of amplicon sequence variants (ASV) in which three different assignment methods were used (Additional file 1: Table S1): (1) IDTaxa using the default conservative threshold of 60, which corresponds to the confidence at which to truncate the output taxonomic classification [49]; (2) assignTaxonomy [46, 50] and (3) BLASTn [51]. The nematode ITS2 rDNA database 1.1.0 [52] containing 9811 complete and non-redundant sequences, with a few additional manual corrections of species assignation corresponding to morphotypes, was used for both IDTaxa and assignTaxonomy. For BLASTn, the three best hits were considered only if they reached a threshold of 90% similarity against NCBI database sequences (2021/04/13). All species-level assignments were ascribed a ‘confidence level' (Additional file 1: Table S1): ﻿*high* *confidence* indicates that all three methods assigned to the same species and *moderate confidence* indicates that two out of the three methods agree at the species level and also the third method was in agreement but could only be assigned to the genus level. Taxonomic assignment was limited to the genus level when there was disagreement across any of the three methods at the species level (but consistency at the genus level) or if there was no similar sequence on GenBank with > 98% of similarity and a query cover > 85% with the corresponding ASV.

The raw ASV data set was curated following a procedure developed by Taberlet et al. (2018). ASVs observed only once in the whole data set were considered spurious and were removed [53]. To delete potential contaminants [54], we discarded any ASVs more abundant in the negative controls (maximum 55 reads) than in other samples and reads with < 0.13% (maximizing the removal of sequences with low abundance in controls) of the total ASV abundance in the entire data set. We only kept ASVs identified as gastrointestinal nematodes at least to the genus level. Finally, any samples with < 1000 reads were removed.

### Phylogeny

A phylogenetic analysis of ASV was conducted using a maximum likelihood tree with the HKY + G [55] model according to “ModelTest” function [56, 57]. We used FigTree 1.4.4 [58] to visualize ASV phylogeny. Although the generation of ASVs enables genetic diversity to be characterized to the highest resolution, the biological relevance of much of this diversity remains unknown. A limitation of using ASVs to characterize genetic diversity is that large sample sizes are required to detect significant statistical differences among samples (sensitive to type II error). To overcome this hurdle, we also clustered ASVs into “taxa” and OTUs (operational taxonomic units) using the “IdCluster” function of the package DECIPHER [59] and a posteriori cutoff of 0.03.

### Statistical analyses on measures of parasitic community

We removed the lungworm *Varestrongylus* from the data set because the focus of the present study was on gastrointestinal nematodes. To control the representativeness of the data, we drew the accumulation curves for the ASV and taxa with the “exact” method of the function “specaccum” of the package vegan [60]. Instead of the exact read count per sample and items, we used the relative frequencies of reads. The read relative frequencies in samples were plotted with ggtree [61]. After controlling for the year, the capture date, the epg and the number of L3 used for DNA extraction, we assessed the effects of age (i.e. young [< 1 year] or adults) and sex (i.e. males or females) on two proxies of diversity of gastrointestinal nematodes community: alpha (measure of community diversity within individual samples) and beta (measure of comparison of dissimilarity between each pair of samples). We measured﻿ the beta diversity by using the Bray-Curtis dissimilarity matrices and the alpha diversity by computing three indices: richness (the number of different items), Shannon-Weaver index [62] and Simpson index (1-D) [63] indices.

To test for differences in nemabiome among individual hosts of different age, sex and site/sector of Chizé, we tested for the influence of these factors, and their interactions, on the alpha and beta diversity. We ensured that all samples come from a unique individual to avoid individual effect in statistical analyses. Doing so, we removed 19 samples (17 from Chizé and 2 from Trois Fontaines; Additional file 1: Figure S2), selecting for each repeated measure the samples with the higher mass of feces used for the coprocultures. Age and sex effects were first assessed at broad scale with the two sites (Chizé and Trois Fontaines) and, second, at the local scale, comparing the three sectors of Chizé (rich coppices, medium coppices and poor beech stand). We used generalized linear models in a model selection approach to test the effects of site, sex and age of individuals on the alpha diversity, with a Poisson family for richness, a Gaussian family for Shannon and a Gaussian (with logit link because Simpson index ranges from 0 to 1) family for Simpson. For the beta diversity, we used a custom function to compute Akaike’s information criterion corrected for small sample size (AICc) based on residual sums of squares [64]. Following the recommendations of Burnham and Anderson [65], we identified models that are biologically meaningful and considering limits due to the sample size. It resulted in 18 competitive models. We then ranked the models for each set of candidate models using the AICc and calculated ΔAICc and AICc weights. We selected the model with the lowest AICc value. Models with ΔAICc ≤ 2 were considered equivalent [65], and in this case, we considered the most parsimonious one, i.e. the model with the lowest degree of freedom.

Differences in nemabiome among samples (beta diversity) were visualized on two dimensions with a non-metric multidimensional scaling (NMDS). We used specifically the NMDS because Bray-Curtis dissimilarities are non-Euclidean embeddable distances [66]. We verified the consistency of patterns between samples resulting from the three taxonomic levels with Procrustes analyses.

## Results

### Parasite sampling

ITS2 rDNA nemabiome analyses were performed on 149 samples (*n* = 36 and 113 samples in Trois Fontaines and Chizé, respectively; Table 1, Fig. 1). The year of sampling had no influence on the nemabiome (Adonis test and Kruskal-Wallis rank sum test; see Additional file 1: Table S2), and this factor was therefore not considered in the following analyses.

**Table 1:** Summary of samples collected in the sites of Chizé and Trois Fontaines in 2018 and 2019

		2018		2019		Total (*N* = 149)
		Chizé (*N* = 57)	Trois Fontaines (*N* = 22)	Chizé (*N* = 56)	Trois Fontaines (*N* = 14)	
Sex	F: female; M: male	32 F 25 M	10 F 12 M	26 F 30 M	5 F 9 M	73 F 76 M
Age class	Y: young; A: adult	11 Y 46 A	13 Y 9 A	17 Y 39 A	5 Y 9 A	46 Y 103 A
Eggs per gram	Median [min–max]	15 [0–270]	7.5 [0–60]	7.5 [0–90]	11.25 [7.5–180]	7.5 [0–270]
Number of L3	Median [min–max]	156 [10.75–1,250]	69.3 [18–8,650]	69.5 [10–819]	51 [10–519]	80 [10–8,650]
Reads	Total	630,926	320,112	555,517	156,868	1,663,423
	Median [min–max]	11,315 [1,638–22,744]	14,308 [1,595–33,527]	9,855.5 [2,749–19,837]	10,288 [2,436–24,330]	10,631 [1,595–33,527]
ASV	Total	53	47	55	33	100
	Median [min–max]	9 [3–16]	12 [6–17]	9 [4–17]	9 [2–19]	9 [2–19]
Taxa	Total	9	11	11	11	16
	Median [min–max]	4 [2–6]	5 [3–7]	4 [2–7]	4 [1–7]	4 [1–7]

**Fig. 2:**
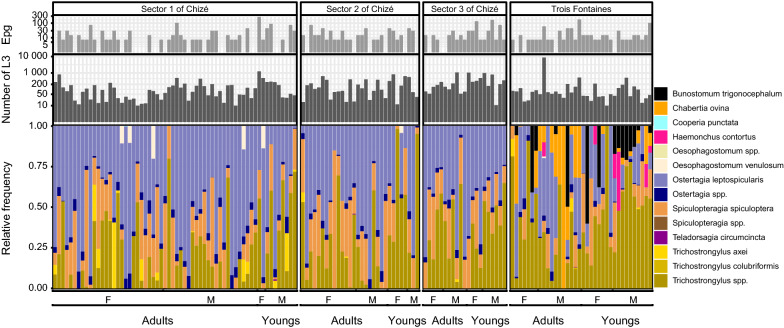
Egg count, number of L3 and read relative frequencies of gastrointestinal nematodes at taxa level. Each stacked bar chart represents the species composition of 149 roe deer samples within which each taxon is defined by one color. The numbers of L3 and fecal egg counts are displayed above each bar chart. The data are split based on site location (Chizé or Trois Fontaines), age (young or adult roe deer) and sex (F: females and M: males). In addition, samples from Chizé are subdivided according to the three different sectors: sector 1: “rich coppices;” sector 2: “medium coppices;” sector 3: “poor beech stand”

The median number eggs per gram of feces was low and heterogeneous (7.5 eggs/g [0; 114]_95%IQR_) as well as the number of L3 used for DNA extraction (80 [12; 993]_95%IQR_; Table 1, Fig. 2). Both had no significant effects on alpha and beta diversity (Pearson correlation test and Adonis test; see Additional file 1: Table S2).

### Taxonomic and community diversity

Considering the whole data set, we detected 100 ASVs resolved across 14 taxa of gastrointestinal nematodes and 2 taxa of lungworms (Table 1 and Additional file 1: Table S1). ASVs were identified either at the genus (43%) or species (57%) level (Additional file 1: Table S1). Ten genera, including ten species, were identified, and their phylogeny was consistent with previous knowledge on nematode taxonomy (Additional file 1: Figure S3). Sampling captured most of the taxa as the taxa accumulation curves rapidly reached a plateau (Additional file 1: Figure S4). Forty-seven ASVs were found only in one sample for which we were not able to disentangle whether their origin was intra-specific genetic diversity or PCR/sequencing error, but following Callahan et al. [46], we considered those ASVs in analyses whatever their frequencies in the data set were. Procrustes analyses performed on ASV with taxa have tight correlations (*P* = 0.001). Thus, we only show results at the ASV taxonomic level, but results from taxa and OTUs are available in Additional file 1: Table S3–S4, Figure S5 and Additional file 2, respectively.

**Fig. 3:**
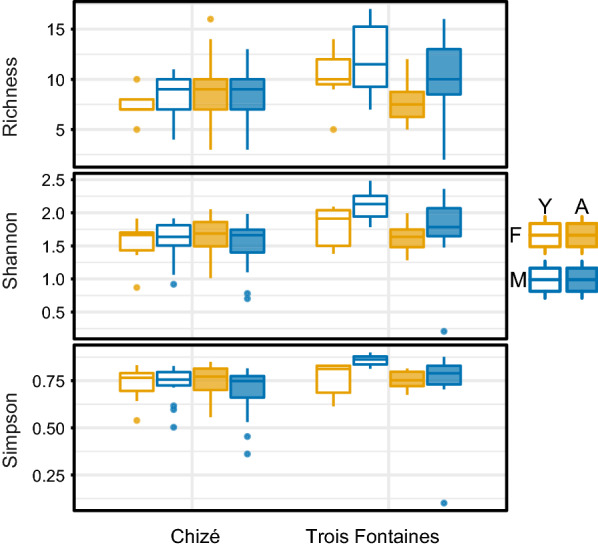
Box plot of richness, Shannon and Simpson (1-D) index calculated on ASVs. Each box plot represents the values of alpha diversity index measured by observed ASV in 130 roe deer samples. Each box includes the 25th and 75th percentiles, the median represented by the line within the box, the whiskers indicating the 10th and 90th percentiles, and points above and under each box are outliers. Box plots are distributed based on factors to compare the alpha diversity corresponding to roe deer of different ages (Y: young, A: adult) and sex (M: male, F: female) in Chizé and Trois Fontaines

According to the best models on the data subset without repeated measures (Table 2; Additional file 1: Table S3), the richness and Simpson diversity index were higher in Trois Fontaines than in Chizé (estimate ± standard error, richness: *β* = 0.20 ± 0.06, *P* = 0.002; Simpson: *β* = 0.24 ± 0.12, *P* = 0.041). The Shannon index, which considers the relative abundances of ASV, included significant interactions between age and site, indicating a higher alpha diversity in younger roe deer in Trois Fontaines (*β* = 0.32 ± 0.13, *P* = 0.017) (Fig. 3; Table 2). At the local scale of Chizé, no differences among the three sectors were detected for all alpha diversity indexes (Fig. 2).

**Table 2:** Best generalized linear models selected for ASVs and each diversity index (richness, Simpson, Shannon)

Diversity index	Best generalized models selected	Variables	Parameter estimate ± SE	*z*-val. or *t*-val	*P*
Richness	$\alpha \sim site$	Intercept	2.14 ± 0.04	60.91	***
		SiteTF	0.20 ± 0.06	3.10	**
Simpson	$\alpha \sim site$	Intercept	1.01 ± 0.05	18.36	***
		SiteTF	0.24 ± 0.12	2.06	*
Shannon	$\alpha \sim age+site+age\times site$	Intercept	1.60 ± 0.04	41.85	***
		SiteTF	0.09 ± 0.09	1.01	–
		AgeY	– 0.02 ± 0.07	– 0.30	–
		AgeY:siteTF	0.32 ± 0.13	2.41	*

Gaussian families were used for Simpson and Shannon regression and Poisson family for richness. The effect of site (Chizé as reference), age (adult as reference) and sex (females as reference) and the interaction between them are reported when included in the selected models. Parameter estimates with SE are reported with the corresponding *z*-value (Poisson family) or *t*-value (Gaussian family) and *P*-value. Statistical significance is represented by *P < 0.05, **P < 0.01 and ***P < 0.001. TF: Trois Fontaines; Y: young

**Fig. 4:**
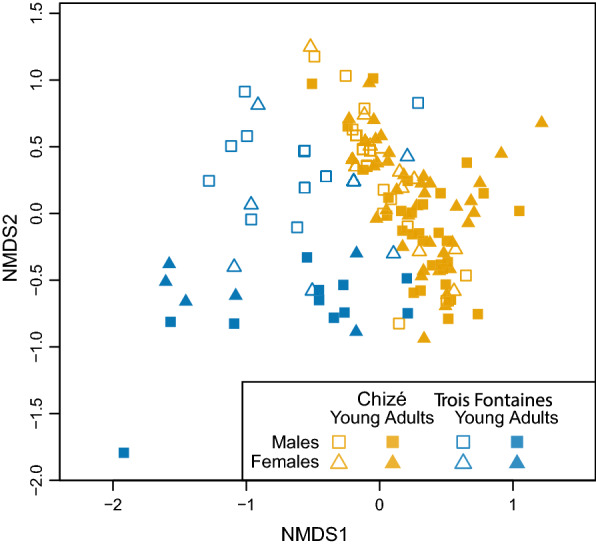
Non-metric multidimensional scaling (NMDS) of nemabiome based on 89 ASVs from the 130 roe deer. The distance between points on ordination represent the Bray-Curtis distance quantifying the dissimilarity between 130 roe deer nemabiomes. Age (young and adult), sex (male and female) and site (Chizé and Trois Fontaines) are defined by points of different colors and shapes. The stress has a value of 0.14

According to the most parsimonious model selected for the Bray-Curtis dissimilarity, the differences of nemabiome between samples were mostly explained by the site (*F*_1,127_ = 35.92, *P* = 0.001) and age (*F*_1,127_ = 12.68, *P* = 0.001) variables. Locally in Chizé, the selected model for Bray-Curtis dissimilarity depended only on age (F_1,94_ = 6.36, P = 0.002). Similarly, we observed distinct groups in the NMDS, with partially separated clouds of points for Chizé and Trois Fontaines and a marked separation between young and adult roe deer in Trois Fontaines (Fig. 4).

**Fig. 5:**
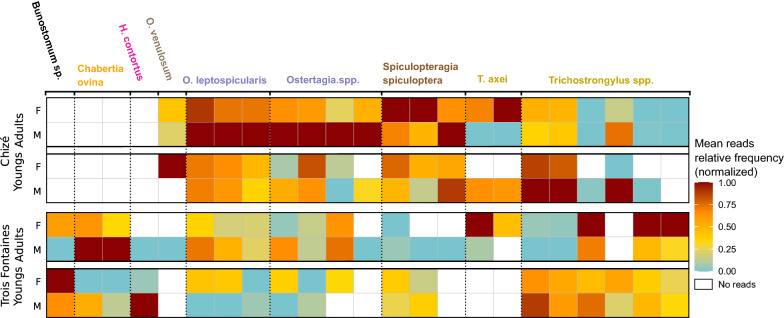
Heatmap of ASVs in samples. Only ASVs found in at least 4% of the 130 samples are displayed. ASV names have the same color when they belong to the same genus. The data were averaged based on site location (Chizé or Trois Fontaines), age (young or adult roe deer) and sex (F: female and M: male) and correspond to each raw of the heatmap. The color indicates the normalized mean read relative frequency of each ASV. Empty cells (white color) correspond to 0 reads at the end of the data curation

The most common species or genera in the entire data set were *Ostertagia leptospicularis*, *Trichostrongylus* spp. and *Spiculopteragia spiculoptera* present in 139, 121 and 120 samples of roe deer out of 149 (93%, 81% and 81%, respectively) with a mean relative abundance among those samples of 44%, 29% and 17%, respectively (Fig. 2). In Chizé, *O. leptospicularis*, *Trichostrongylus* spp. and *S. spiculoptera* were present in 98%, 71% and 79% of samples in the sector 1; 93%, 80% and 90% in the sector 2; and 100%, 86% and 100% in the sector 3. The mean relative abundances of species among those samples were 53%, 22% and 17% (sector 1), 46%, 28% and 23% (sector 2) and 45%, 33% and 19% (sector 3). In Trois Fontaines, *O. leptospicularis*, *Trichostrongylus* spp. and *S. spiculoptera* were present in 81%, 97% and 64% of samples with a mean relative abundance among those samples of 24%, 42% and 9%. The ovine species *Chabertia ovina*, *Haemonchus contortus* and *Bunostomum* sp. were only detected in Trois Fontaines (Figs. 2, 5), explaining the significantly higher alpha diversity in Trois Fontaines (Table 2) and the significative difference of nemabiome between the two sites (Table 3). We also observed variation in the prevalence and relative abundance of five *Trichostrongylus* spp. ASVs between the two sites and between the host age groups, with greater differences of abundance between age in Trois Fontaines compared to Chizé (Fig. 5, A11), as indicated by the significant interaction between site and age on Shannon diversity index.

**Table 3:** PerMANOVA model selected for ASVs and Bray-Curtis dissimilarities matrix

Diversity index	Best generalized models selected	Variables	*R*^2^	*F*-value	*P*
Bray-Curtis	$\beta \sim site+age$	Residuals	0.72	–	–
		Site	0.20	35.92	***
		Age	0.07	12.68	***

The effects of site, age, sex and the interaction between them are reported when included in the selected model. Statistical significance is represented by ***P < 0.001

## Discussion

ITS2 rDNA nemabiome metabarcoding provided fine resolution data on the gastrointestinal nematode parasite community composition of two populations of roe deer in France. The gastrointestinal nematode species detected in Trois Fontaines and Chizé correspond largely to species previously detected on necropsies of roe deer in the two study sites [13, 67]. We also detected species not previously described in these two populations [13, 67], but previously found in other roe deer populations (e.g. in Spain [37] and in the Netherlands [36]). This study provided new insights into site- and age-specific gastrointestinal nematode diversity because we showed that young roe deer have different nemabiomes compared to adults, with more pronounced effects of age in one site (i.e. Trois Fontaines).

### Diversity of gastrointestinal nematodes in roe deer

In accordance with previous studies on helminths of roe deer (e.g. [37, 38]), the main species detected were *O. leptospicularis, Trichostrongylus* spp. and *Spiculopteragia* spp. These gastrointestinal nematodes are commonly found in roe deer, confirming our hypothesis H1 that the dominant nematode species were roe deer-specific parasites given that these populations are enclosed in fenced forest areas. However, we found some differences between the two study sites. We have detected generalist species (i.e. gastrointestinal nematodes species found in different host species) at a relatively high frequency in Trois Fontaines but not in Chizé. In addition to *C. ovina* previously detected in Trois Fontaines [13], we have detected for the first time the presence of the highly pathogenic species *H. contortus* and *B. trigonocephalum* in this population. In Chizé and Trois Fontaines, we noted the presence of *T. axei* and *O. venulosum*, at low relative abundance, also considered as generalist species, and *T. colubriformis* (only in Chizé) and *T. circumcincta*, which generally infect sheep and goats [30, 68]. The identifications of *H. contortus* and *T. axei* are at a “high confidence level" because these species were well represented in the nemabiome database and the three different methods of assignment provided the same species assignment. For *Bunostomum* sp., ASVs were only assigned at the genus level as a result of a relatively low level of identity (92% over 283 bp [query cover of 100%, e-value = 9e^−110^]) to the best BLAST hit in GenBank (Additional file 1: Table S1). To our knowledge, *B. trigonocephalum* of sheep and *B. phlebotomum* of calves are the only *Bunostomum* species which have been identified in roe deer in Europe (e.g. [36]). These two species are highly divergent, and considering the low level of intra-specific variation observed within *B. trigonocephalum* [69], it is possible that the *Bunostomum* sp. identified in Trois Fontaines is a previously unidentified, or cryptic, species closely related to *B. trigonocephalum*.

For some ASVs, the taxonomic identification did not reach the species level, e.g. *Trichostrongylus* spp. (67% among this genus), *Spiculopteragia* spp. (6%) or *Ostertagia* spp. (67%). This may be largely due to the lack of reference sequence available for these species in the nemabiome database and ultimately in GenBank. This issue is accentuated when applying nemabiome metabarcoding to wildlife hosts compared with domestic livestock where parasite communities are better defined. For example, there is just a single ITS2 reference sequence for *Trichostrongylus capricola* compared to 299 ITS2 reference sequences for *Haemonchus contortus* in the nemabiome database (see also Aivelo and Medlar 2018 for the SILVA database). The potential presence of cryptic species and/ or hybrids of closely related species [70] may further complicate accurate species assignation. Consequently, the generation of reference sequences from morphologically validated parasites should continue to be a research priority for the scientific community as a whole.

*Ashworthius sidemi* was not detected in Trois Fontaines, although it has previously been isolated on roe deer carcasses during winter in this study area [13, 71]. This may be due to the hypobiosis in this species [72] or to the fact that during winter females contained only non-embryonated eggs [73]. Further investigations on the seasonal nemabiome will be required to determine the complete gastrointestinal nematode species richness and variations during the year and species.

### Effects of site, age and sex class on the nemabiome

In Trois Fontaines, the age of individuals had a major influence on the gastrointestinal nematode species community found in roe deer, with a higher alpha diversity (*β* = 0.32 ± 0.13, *P* = 0.017) observed in young compared to adult animals in accordance with our prediction (H2). This age effect on beta diversity is mostly driven by some specific variants of *Trichostrongylus* spp. in both sites and of *H. contortus* in Trois Fontaines that are more prevalent, and of higher relative abundance, in young roe deer compared to adults. The limited influence of age on prevalence and relative abundance observed for some of the other parasite species (see also e.g. [74]) may result from survival mechanisms of these parasite species, such as the dysregulation of the immune response of the host [75], from an already developed and efficient immune response of the host when young roe deer were captured against these parasite species (i.e. at 8–10 months of age in our study) or from an immune response that is inefficient.

The higher prevalence and relative abundance of some gastrointestinal nematode species found in adults in Chizé but not in adults in Trois Fontaines suggest a higher ability to resist to certain parasite species (e.g. some *Trichostrongylus* variants) in adult roe deer in Trois Fontaines. There is probably a synergistic interaction among the body condition of roe deer, their immune response and the richness of parasites to which they have been exposed [11]. Individuals in lower body condition are more susceptible to parasite infection or/and high levels of parasitism are suspected to negatively impact body condition [19]. Such negative correlation between physiological performance and parasitic burden has previously been observed to be more pronounced in Chizé than in Trois Fontaines [76]. In addition, in Trois Fontaines, the higher body condition of roe deer [10] and strongyle species diversity may have allowed roe deer to invest in innate immunity [77], which could prevent the establishment or facilitate the expulsion of strongyles [78]. In contrast, roe deer in Chizé, exposed to a lower species richness of strongyles and being in lower body condition, invest more in adaptive response, which is considered less costly than innate response [79].

Contrary to our expectation (H3), the alpha diversity of the parasite communities did not differ between males and females in this study. Indeed, the probability of exposure to gastrointestinal species is equal between males and females as they do not spatially segregate [80], and few sexual differences in their feeding behavior or activity have been observed between males and females [81, 82]. In addition, no sex-specific physiological status occurred during the study period, such as nursing young [4] or rutting [83], that could have diverted or limited the allocation of resources to parasite defenses. Sampling during such sex-specific energetically costly periods could be pertinent to consider the mechanisms underlying sex and age differences in gastrointestinal nematodes communities.

Despite the differences in habitat quality, the nemabiome of roe deer was not different among the three sectors of Chizé (H4). This suggests that local ecological variation and the resulting differences in body conditions among roe deer inhabiting these different habitats [40] do not affect the nemabiome. This may also be explained by the movement of roe deer among sectors that can spread the parasites among the feeding areas [40, 84].

### Detection of parasite species generally associated with domestic ungulates

The occurrence in roe deer of Trois Fontaines of parasites commonly detected in small domestic ruminants, including highly pathogenic species (e.g. *H. contortus*, *B. trigonocephalum*), confirmed previous studies showing that wildlife can share some parasites found in livestock (e.g. [33, 38]). To our knowledge, only four to six domestic sheep have been bred for several years on the other side of a road surrounding the fenced study site of Trois Fontaines (C. Warnant, personal communication). Even if the fence of the study site is regularly checked for damages, the fence does not totally prevent a few roe deer from leaving and/or entering the reserve (e.g. after the Lothar hurricane in 1999). Such movements may have facilitated the introduction of generalist parasites in the population of Trois Fontaines. The consequences of these pathogenic species on roe deer fitness remain however to be determined.

## Conclusion

In the present study we investigated the community of gastrointestinal nematode species in two isolated populations of roe deer using ITS2 rDNA nemabiome metabarcoding. Most gastrointestinal nematodes detected in the two sites were parasites classically found in roe deer. Even though the two populations inhabit fenced areas, we also observed gastrointestinal nematodes usually found in domestic ungulates in one of the populations (Trois Fontaines). Despite a fence restraining movements of animals from and to the study site, migrations of a few individuals may have contributed to the movements of parasites among populations, including generalist parasites from domestic ungulates. This could have important implications for the management of wild populations and the introduction of non-native species to wildlife communities. Therefore, it appears highly relevant to study roe deer and domestic ungulates living in sympatry in anthropogenic landscapes dominated by farmland to better understand the dynamic of gastrointestinal nematodes among wild and domestic populations and the risk of cross-transmission. This work also illustrated the power and limitations of ITS2 nemabiome metabarcoding for wildlife studies. It allows the screening of large numbers of samples for a large number of parasites, with identification to the species level in many cases. However, some parasites could still only be identified to the genus level emphasizing the need to continue to enrich the DNA sequence databases for more comprehensive identification.

## Supplementary Information

**Additional file 1: Figure S1.** Flowchart summarizing the data curation from Illumina amplicon fastq files to the final curating dataset. **Table S1.** Taxonomic assignment of ASVs using a combination of three separate methods. **Figure S2.** Read relative frequencies of GIN at taxa level in samples of 19 recaptured roe deer. **Table S2.** Results of statistical tests of the relation between diversity index and year of samples, number of L3 and epg. **Figure S3.** Unrooted maximum likelihood tree of ASVs in samples. **Figure S4.** Sample accumulation curves. **Table S3.** Set of generalized linear models and perMANOVA models sorted by AICc value. **Table S4.** Generalized linear models and perMANOVA models selected for taxa. **Figure S5.** Non-metric multidimensional scaling (Taxa) of nemabiome.

**Additional file 2. **OTUs analysis.

## Data Availability

The roe deer samples information and the sequence data generated and analyzed during the current study are available in the NCBI Short Read Archive under project accession number PRJNA765944 (SUB9936230).

## Abbreviations

ITS2: Internal transcribed spacer 2
ASV: Amplified sequence variant
epg: Eggs per gram

## References

[1] Hoberg EP, Kocan AA, Rickard LG, William SM, Pybus MJ, Kocan AA (2001). Gastrointestinal strongyles in wild ruminants. Parasitic diseases of wild mammals.

[2] Roeber F, Jex AR, Gasser RB (2013). Impact of gastrointestinal parasitic nematodes of sheep, and the role of advanced molecular tools for exploring epidemiology and drug resistance—an Australian perspective. Parasites Vectors.

[3] Craig BH, Pilkington JG, Pemberton JM (2006). Gastrointestinal nematode species burdens and host mortality in a feral sheep population. Parasitology.

[4] Leivesley JA, Bussière LF, Pemberton JM, Pilkington JG, Wilson K, Hayward AD (2019). Survival costs of reproduction are mediated by parasite infection in wild Soay sheep. Ecol Lett.

[5] Kelehear C, Brown GP, Shine R (2011). Influence of lung parasites on the growth rates of free-ranging and captive adult cane toads. Oecologia.

[6] Tompkins DM, Wilson K (1998). Wildlife disease ecology: from theory to policy. Trends Ecol Evol.

[7] Gunn A, Irvine RJ (2003). Subclinical parasitism and ruminant foraging strategies—a review. Wildl Soc Bull.

[8] Thompson RCA, Lymbery AJ, Smith A (2010). Parasites, emerging disease and wildlife conservation. Int J Parasitol.

[9] Lochmiller RL, Deerenberg C (2000). Trade-offs in evolutionary immunology: just what is the cost of immunity?. Oikos.

[10] Gilot-Fromont E, Jégo M, Bonenfant C, Gibert P, Rannou B, Klein F (2012). Immune phenotype and body condition in roe deer: individuals with high body condition have different, not stronger immunity. PLoS ONE.

[11] Beldomenico PM, Begon M (2010). Disease spread, susceptibility and infection intensity: vicious circles?. Trends Ecol Evol.

[12] Tompkins DM, Dunn AM, Smith MJ, Telfer S (2011). Wildlife diseases: from individuals to ecosystems. J Anim Ecol.

[13] Body G, Ferté H, Gaillard J-M, Delorme D, Klein F, Gilot-Fromont E (2011). Population density and phenotypic attributes influence the level of nematode parasitism in roe deer. Oecologia.

[14] Aleuy OA, Ruckstuhl K, Hoberg EP, Veitch A, Simmons N, Kutz SJ (2018). Diversity of gastrointestinal helminths in Dall’s sheep and the negative association of the abomasal nematode, *Marshallagia marshalli*, with fitness indicators. PLoS ONE.

[15] Sinclair R, Melville L, Sargison F, Kenyon F, Nussey D, Watt K (2016). Gastrointestinal nematode species diversity in Soay sheep kept in a natural environment without active parasite control. Vet Parasitol.

[16] Gruver AL, Hudson LL, Sempowski GD (2007). Immunosenescence of ageing. J Pathol.

[17] Peters A, Delhey K, Nakagawa S, Aulsebrook A, Verhulst S (2019). Immunosenescence in wild animals: meta-analysis and outlook. Ecol Lett.

[18] Budischak SA, O’Neal D, Jolles AE, Ezenwa VO (2018). Differential host responses to parasitism shape divergent fitness costs of infection. Funct Ecol.

[19] Bourgoin G, Portanier E, Poirel M-T, Itty C, Duhayer J, Benabed S (2021). Reproductive females and young mouflon (*Ovis gmelini musimon* × *Ovis* sp.) in poor body condition are the main spreaders of gastrointestinal parasites. Parasitology.

[20] Hayward AD (2013). Causes and consequences of intra- and inter-host heterogeneity in defence against nematodes. Parasite Immunol.

[21] Klein SL (2000). The effects of hormones on sex differences in infection: from genes to behavior. Neurosci Biobehav Rev.

[22] Moore SL, Wilson K (2002). Parasites as a viability cost of sexual selection in natural populations of mammals. Science.

[23] Markle JG, Fish EN (2014). SeXX matters in immunity. Trends Immunol.

[24] Froeschke G, Harf R, Sommer S, Matthee S (2010). Effects of precipitation on parasite burden along a natural climatic gradient in southern Africa—implications for possible shifts in infestation patterns due to global changes. Oikos.

[25] Cheynel L, Lemaître J-F, Gaillard J-M, Rey B, Bourgoin G, Ferté H (2017). Immunosenescence patterns differ between populations but not between sexes in a long-lived mammal. Sci Rep.

[26] Davidson RK, Ličina T, Gorini L, Milner JM (2015). Endoparasites in a Norwegian moose (Alces alces) population—Faunal diversity, abundance and body condition. Int J Parasitol Parasites Wildl.

[27] Oliver-Guimerá A, Martínez-Carrasco C, Tvarijonaviciute A, Ruiz de Ybáñez MR, Martínez-Guijosa J, López-Olvera JR (2017). The physiological cost of male-biased parasitism in a nearly monomorphic mammal. Parasites Vectors..

[28] Dobson A, Lafferty KD, Kuris AM, Hechinger RF, Jetz W (2008). Homage to Linnaeus: how many parasites? How many hosts?. Proc Natl Acad Sci USA.

[29] Goater TM, Goater CP, Esch GW (2013). Parasitism: the diversity and ecology of animal parasites.

[30] Taylor MA, Coop RL, Wall RL (2015). Veterinary parasitology.

[31] Aivelo T, Medlar A (2018). Opportunities and challenges in metabarcoding approaches for helminth community identification in wild mammals. Parasitology.

[32] Avramenko RW, Redman EM, Lewis R, Yazwinski TA, Wasmuth JD, Gilleard JS (2015). Exploring the gastrointestinal “Nemabiome”: deep amplicon sequencing to quantify the species composition of parasitic nematode communities. PLoS ONE.

[33] Barone CD, Wit J, Hoberg EP, Gilleard JS, Zarlenga DS (2020). Wild ruminants as reservoirs of domestic livestock gastrointestinal nematodes. Vet Parasitol.

[34] Obanda V, Maingi N, Muchemi G, Ng’ang’a CJ, Angelone S, Archie EA (2019). Infection dynamics of gastrointestinal helminths in sympatric non-human primates, livestock and wild ruminants in Kenya. PLoS ONE.

[35] Walker JG, Morgan ER (2014). Generalists at the interface: nematode transmission between wild and domestic ungulates. Int J Parasitol Parasites Wildl.

[36] Borgsteede FHM, Jansen J, Van Nispen tot Pannerden HPM, Van Der Burg WPJ, Noorman N, Poutsma J (1990). Untersuchungen über die helminthen-fauna beim reh (*Capreolus capreolus* L.) in den Niederlanden. Z Jagdwiss.

[37] Pato FJ, Vázquez L, Díez-Baños N, López C, Sánchez-Andrade R, Fernández G (2013). Gastrointestinal nematode infections in roe deer (*Capreolus capreolus*) from the NW of the Iberian Peninsula: assessment of some risk factors. Vet Parasitol.

[38] Zaffaroni E, Teresa Manfredi M, Citterio C, Sala M, Piccolo G, Lanfranchi P (2000). Host specificity of abomasal nematodes in free ranging alpine ruminants. Vet Parasitol.

[39] Segonds-Pichon A, Ferté H, Gaillard J-M, Lamarque F, Duncan P (2000). Nematode infestation and body condition in roe deer (*Capreolus capreolus*). Game Wildl Sci.

[40] Pettorelli N, Gaillard J-M, Duncan P, Maillard D, Van Laere G, Delorme D (2003). Age and density modify the effects of habitat quality on survival and movements of roe deer. Ecology.

[41] Gaillard J-M, Duncan P, Delorme D, Van Laere G, Pettorelli N, Maillard D (2003). Effects of hurricane Lothar on the population dynamics of European roe deer. J Wildl Manag.

[42] Gaudry W, Gaillard J-M, Saïd S, Bonenfant C, Mysterud A, Morellet N (2018). Same habitat composition but different use: evidence of context-dependent habitat selection in roe deer females. Sci Rep.

[43] Gaillard J-M, Delorme D, Boutin J-M, Van Laere G, Boisaubert B, Pradel R (1993). Roe deer survival patterns: a comparative analysis of contrasting populations. J Anim Ecol.

[44] O’Connor LJ, Walkden-Brown SW, Kahn LP (2006). Ecology of the free-living stages of major trichostrongylid parasites of sheep. Vet Parasitol.

[45] Raynaud J-P, William G, Brunault G (1970). Etude de l’efficacité d’une technique de coproscopie quantitative pour le diagnostic de routine et le contrôle des infestations parasitaires des bovins, ovins, équins et porcins. Ann Parasitol Hum Comp.

[46] Callahan BJ, McMurdie PJ, Rosen MJ, Han AW, Johnson AJA, Holmes SP (2016). DADA2: High-resolution sample inference from Illumina amplicon data. Nat Methods.

[47] Martin M (2011). Cutadapt removes adapter sequences from high-throughput sequencing reads. EMBnet J.

[48] Taberlet P, Bonin A, Zinger L, Coissac E. Environmental DNA: for biodiversity research and monitoring. Oxford: Oxford University Press; 2018.

[49] Murali A, Bhargava A, Wright ES (2018). IDTAXA: a novel approach for accurate taxonomic classification of microbiome sequences. Microbiome.

[50] Wang Q, Garrity GM, Tiedje JM, Cole JR (2007). Naïve bayesian classifier for rapid assignment of rRNA sequences into the new bacterial taxonomy. Appl Environ Microbiol.

[51] Altschul SF, Gish W, Miller W, Myers EW, Lipman DJ (1990). Basic local alignment search tool. J Mol Biol.

[52] Workentine ML, Chen R, Zhu S, Gavriliuc S, Shaw N, de Rijke J (2020). A database for ITS2 sequences from nematodes. BMC Genet.

[53] Callahan BJ, McMurdie PJ, Rosen MJ, Han AW, Johnson AJA, Holmes SP (2016). DADA2: High resolution sample inference from Illumina amplicon data. Nat Methods.

[54] Schnell IB, Bohmann K, Gilbert MTP (2015). Tag jumps illuminated—reducing sequence-to-sample misidentifications in metabarcoding studies. Mol Ecol Resour.

[55] Hasegawa M, Kishino H, Yano T (1985). Dating of the human-ape splitting by a molecular clock of mitochondrial DNA. J Mol Evol.

[56] Posada D, Crandall KA (1998). modeltest: testing the model of DNA substitution. Bioinformatics.

[57] Schliep KP (2011). phangorn: phylogenetic analysis in R. Bioinformatics Oxford Academic.

[58] Rambaut A. (2014). FigTree v1.4.4. http://tree.bio.ed.ac.uk/software/figtree/. Accessed 4 Dec 2020.

[59] Wright ES (2016). Using DECIPHER v2.0 to analyze big biological sequence data in R. R J.

[60] Oksanen J, Blanchet FG, Friendly M, Roeland K, Legendre P, McGlinn D, et al. (2020). vegan: Community Ecology Package. R package v2.5–7. https://CRAN.R-project.org/package=vegan.

[61] Yu G, Smith DK, Zhu H, Guan Y, Lam TTY (2017). ggtree: an R package for visualization and annotation of phylogenetic trees with their covariates and other associated data. Methods Ecol Evol.

[62] Shannon CE, Weaver W (1948). A mathematical theory of communication.

[63] Simpson EH (1949). Measurement of diversity. Nature.

[64] Dyson K. Custom community ecology helper R scripts [Internet]. 2018. https://github.com/kdyson/R_Scripts

[65] Burnham KP, Anderson DR (2002). Model selection and multimodel inference: a practical information-theoretic approach.

[66] Legendre P, Legendre L (1998). Numerical ecology.

[67] Lienard E, Depaquit J, Ferté H (2006). *Spiculopteragia mathevossiani* Ruchliadev, 1948 is the minor morph of *Spiculopteragia spiculoptera* (Gushanskaya, 1931): molecular evidence. Vet Res.

[68] Wyrobisz-Papiewska A, Kowal J, Nosal P, Chovancová G, Rehbein S (2018). Host specificity and species diversity of the Ostertagiinae Lopez-Neyra, 1947 in ruminants: a European perspective. Parasites Vectors.

[69] Wang C, Gao J-F, Chang QC, Zou FC, Zhao Q, Zhu X-Q (2013). Sequence variability in four mitochondrial genes among *Bunostomum trigonocephalum* isolates from four provinces in China. J Helminthol.

[70] Wyrobisz A, Kowal J, Nosal P (2016). Insight into species diversity of the Trichostrongylidae Leiper, 1912 (Nematoda: Strongylida) in ruminants. J Helminthol.

[71] Lehrter V, Jouet D, Liénard E, Decors A, Patrelle C (2016). *Ashworthius sidemi* Schulz, 1933 and *Haemonchus contortus* (Rudolphi, 1803) in cervids in France: integrative approach for species identification. Infect Genet Evol.

[72] Dróżdż J, Demiaszkiewicz A, Lachowicz J (2003). Expansion of the Asiatic parasite *Ashworthius sidemi* (Nematoda, Trichostrongylidae) in wild ruminants in Polish territory. Parasitol Res.

[73] Vadlejch J, Kyriánová I, Rylková K, Zikmund M, Langrova I (2017). Health risks associated with wild animal translocation: a case of the European bison and an alien parasite. Biol Invasions.

[74] Santín-Durán M, Alunda JM, Hoberg EP, de la Fuente C (2008). Age distribution and seasonal dynamics of abomasal helminths in wild red deer from central Spain. J Parasitol.

[75] Else KJ (2005). Have gastrointestinal nematodes outwitted the immune system?. Parasite Immunol.

[76] Jégo M, Ferté H, Gaillard J-M, Klein F, Crespin L, Gilot-Fromont E (2014). A comparison of the physiological status in parasitized roe deer (*Capreolus capreolus*) from two different populations. Vet Parasitol.

[77] Bordes F, Morand S (2009). Coevolution between multiple helminth infestations and basal immune investment in mammals: cumulative effects of polyparasitism?. Parasitol Res.

[78] Inclan-Rico JM, Siracusa MC (2018). First responders: innate immunity to helminths. Trends Parasitol.

[79] Martin LB, Weil ZM, Nelson RJ (2008). Seasonal changes in vertebrate immune activity: mediation by physiological trade-offs. Philos Trans R Soc Lond B Biol Sci.

[80] Bonenfant C, Gaillard J-M, Dray S, Loison A, Royer M, Chessel D (2007). Testing sexual segregation and aggregation: old ways are best. Ecology.

[81] José CS, Lovari S, Ferrari N (1996). Temporal evolution of vigilance in roe deer. Behav Processes.

[82] Merceron G, Viriot L, Blondel C (2004). Tooth microwear pattern in roe deer (*Capreolus capreolus* L.) from Chizé (Western France) and relation to food composition. Small Rumin Res.

[83] Habig B, Doellman MM, Woods K, Olansen J, Archie EA (2018). Social status and parasitism in male and female vertebrates: a meta-analysis. Sci Rep.

[84] Portanier E, Garel M, Devillard S, Duhayer J, Poirel M-T, Henri H (2019). Does host socio-spatial behavior lead to a fine-scale spatial genetic structure in its associated parasites?. Parasite.

