# Supplementary material for: Metabarcoding in two isolated populations of wild roe deer (Capreolus capreolus) reveals variation in gastrointestinal nematode community composition between regions and among age classes
Source: Parasit Vectors. 2021 Dec 4;14:594. doi: 10.1186/s13071-021-05087-5 (PMC8642965; doi:10.1186/s13071-021-05087-5)
Supplement: Supplementary file 1 — Additional file 1: Figure S1. Flowchart summarizing the data curation from Illumina amplicon fastq files to the final curating dataset. Table S1. Taxonomic assignment of ASVs using a combination of three separate methods. Figure S2. Read relative frequencies of GIN at taxa level in samples of 19 recaptured roe deer. Table S2. Results of statistical tests of the relation between diversity index and year of samples, number of L3 and epg. Figure S3. Unrooted maximum likelihood tree of ASVs in samples. Figure S4. Sample accumulation curves. Table S3. Set of generalized linear models and perMANOVA models sorted by AICc value. Table S4. Generalized linear models and perMANOVA models selected for taxa. Figure S5. Non-metric multidimensional scaling (Taxa) of nemabiome. [file 13071_2021_5087_MOESM1_ESM.docx]

**Metabarcoding in two isolated populations of wild roe deer (*Capreolus capreolus)* reveals variation in gastrointestinal nematode community composition between regions and among age classes**

Camille Beaumelle^1,3^* and Libby Redman^2,^*, Jill de Rijke^2^, Janneke Wit^2^, Slimania Benabed^1,4^, François Debias^1^, Jeanne Duhayer^1^, Sylvia Pardonnet^1^, Marie-Thérèse Poirel^1,4^, Gilles Capron^5^, Stéphane Chabot^5^, Benjamin Rey^1^, Glenn Yannic^3^, John S Gilleard^2,^** and Gilles Bourgoin^1,4,^**

^1^Université de Lyon, Université Lyon 1, CNRS, Laboratoire de Biométrie et Biologie Evolutive UMR 5558, F-69100 Villeurbanne, France

^2^Comparative Biology and Experimental medicine, Host-Parasites Interactions Program, Faculty of Veterinary Medicine, University of Calgary, Calgary, Alberta, Canada.

^3^Université Grenoble Alpes, Université Savoie Mont Blanc, CNRS, LECA, 38000, Grenoble, France

^4^Université de Lyon, VetAgro Sup, Campus Vétérinaire de Lyon, F-69280 Marcy l’Etoile, France

^5^Office Français de la Biodiversité, F-75008 Paris, France

*These authors contributed equally to this work

**Co-senior authors

**Corresponding author:**

E-mail address: beaumelle.camille@gmail.com

**Figure S1.** Flowchart summarizing the data curation from Illumina amplicon fastq files to the final curating dataset.

**
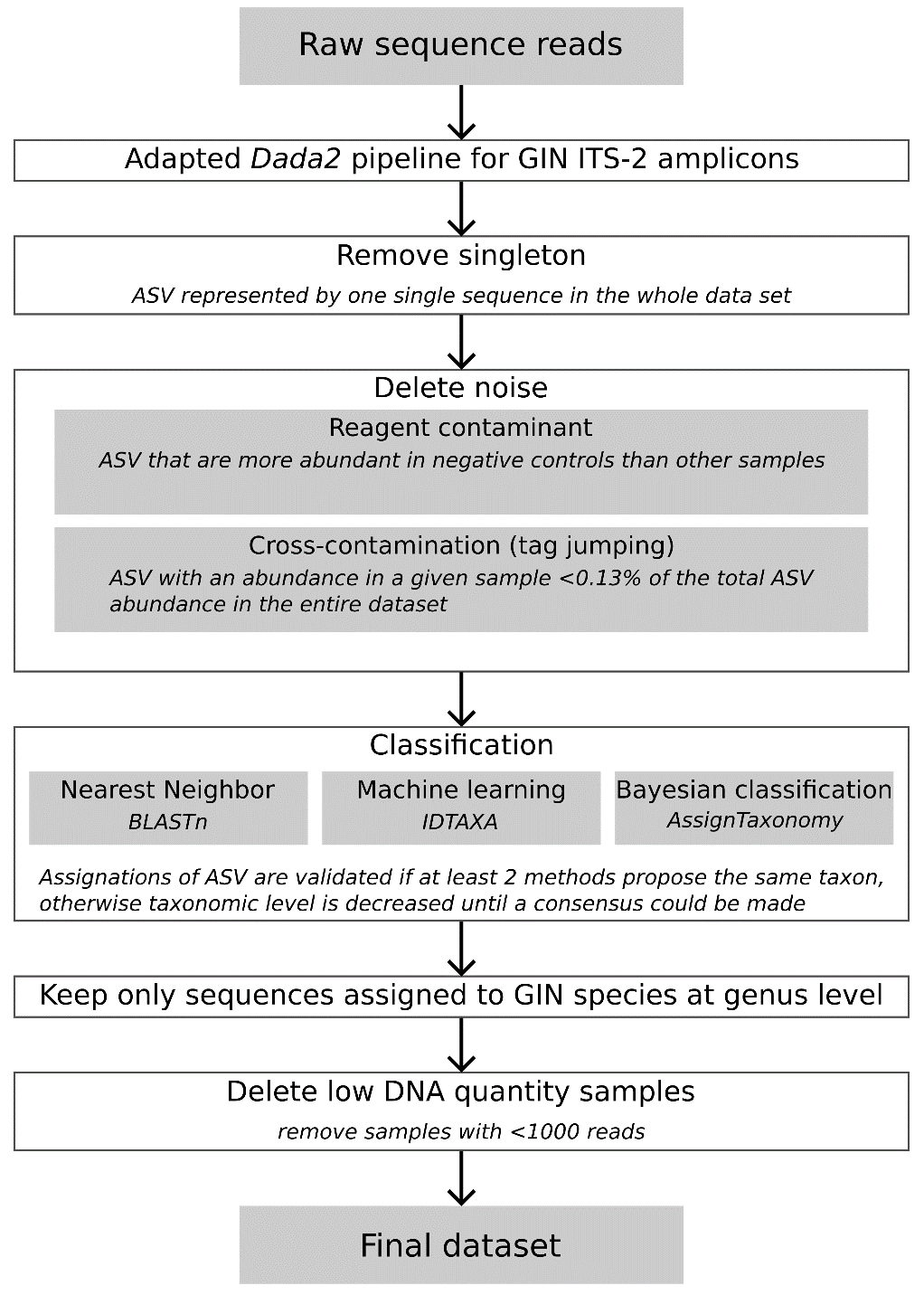
**

**Table S1.** Taxonomic assignment of ASVs observed in two isolated populations of wild roe deer (*Capreolus capreolus)* using a combination of three separate methods. The “Assignment” column indicates the assignment we use in analyses and is based on the consensus agreement of at least two out of the three assignment methods. Assignments that do not reach the genus level are not used in the analyses All species-level assignments have a degree of confidence associated with them: high confidence indicates all 3 methods predict the same species and moderate confidence indicates 2 out of the 3 methods agree at the species level. Where there is disagreement across any of the three methods the next highest taxonomic level is used for assignment i.e. genus.

| IDTaxa 60 | AssignTaxonomy | BLASTn consensus of the 3 best matches with identity >90%  (identity values of the 3 best matches) | Assignment | Confidence level of the assignation | List of ASV |
| --- | --- | --- | --- | --- | --- |
| ***Bunostomum*** |  |  |  |  |  |
| Root | *Bunostomum trigonocephalum* | *Bunostomum trigonocephalum*  *(92.3%-91.9%-91.6%)* | *Bunostomum* |  | ASV 12 |
| Root | *Bunostomum* | Root | Root |  | ASV 134 ASV 139 |
| ***Chabertia*** |  |  |  |  |  |
| *Chabertia ovina* | *Chabertia ovina* | *Chabertia ovina*  *(100%-100%-99.6%)*  *(99.3%-98.6%-98.2%)*  *(99.6%-98.9%-98.9%)*  *(99.3%-99.3%-98.9%)*  *(100%-98.9%-98.9%)*  *(100%-98.9%-98.6%)* | *Chabertia ovina* | high confidence | ASV 14  ASV 36 ASV 70 ASV 72 ASV 73 ASV 88 |
| ***Cooperia*** |  |  |  |  |  |
| *Cooperia punctata* | *Cooperia punctata* | *Cooperia punctata*  *(100%-100%_99.7%)* | *Cooperia punctata* | high confidence | ASV 168 |
| ***Haemonchus*** |  |  |  |  |  |
| *Haemonchus contortus* | *Haemonchus contortus* | *Haemonchus contortus*  *(100%-100%-100%)*  *(100%-100%-96.8%)*  *(99.6%-97.5%-97.2%)*  *(100%-99.3%-99.3%)*  *(98.9%-98.6%-98.6%)*  *(98.6%-98.2%-98.2%)* | *Haemonchus contortus* | high confidence | ASV 7  ASV 10  ASV 26 ASV 32  ASV 20*  ASV 86* |
| ***Oesophagostomum*** |  |  |  |  |  |
| *Oesophagostomum venulosum* | *Oesophagostomum venulosum* | *Oesophagostomum venulosum*  *(99.7%-99.7%-99.4%)* | *Oesophagostomum venulosum* | high confidence | ASV 21 |
| *Oesophagostomum* | Chabertiidae | *Oesophagostomum*  *(96.4%-96.4%-96.4%)* | *Oesophagostomum* |  | ASV 182 |
| ***Ostertagia*** |  |  |  |  |  |
| *Ostertagia leptospicularis* | *Ostertagia* | *Ostertagia leptospicularis*  *(100%-100%-99.7%)*  *(100%-99.7%-99.7%)*  *(99.7%-99.7%-99.3%)*  *(99.7%-99.7%-99.3%)*  *(99.3%-99.3%-99.0%)*  *(100%-100%-99.7%)*  *(99.7%-99.7%-99.3%)*  *(99.3%-99.3%-99.0%).*  *(100%-100%-99.5%)* | *Ostertagia leptospicularis* | moderate confidence | ASV 1  ASV 8 ASV 9  ASV 58 ASV 93 ASV 98  ASV 111 ASV 167 ASV 179 |
| *Ostertagia* | *Ostertagia* | *Ostertagia leptospicularis*  *(99.0%-99.0%-99.3%)*  *(99.7%-99.7%-99.3%)*  *(99.3%-99.3%-99.0%)*  *(99.7%-99.7%-99.3%)*  *(99.7%-99.7%-99.3%)*  *(99.3%-99.3%-99.0%)*  *(99.3%-99.3%-99.0%)*  *(100%-100%-100%)*  *(99.7%-99.7%-99.3%)*  *(91.1%-90.7%-90.7%)*  *(99.3%98.9%-99.3%)*  *(100%-98.6%-100%)*  *(100%-100%-100%)*  *(100%-97.9%-100%)* | *Ostertagia* |  | ASV 15  ASV 53 ASV 55  ASV 63 ASV 87  ASV 106 ASV 108 ASV 154 ASV 161 ASV 174 ASV 181 ASV 203  ASV 206 ASV 225 |
| *Ostertagia* | *Ostertagia gruehneri* | *Ostertagia leptospicularis*  *(99.7%-99.7%-100%)*  *(99.3%-99.3%-99.0%)*  *(99.3%-99.3%-99.0%)*  *(100%-95.7%-100%)*  *(100%-96.9%-100%)* | *Ostertagia* |  | ASV 27 ASV 39 ASV 51  ASV 178  ASV 198 |
| *Ostertagia leptospicularis* | *Ostertagia gruehneri* | *Ostertagia leptospicularis*  *(99.7%-99.7%-99.3%)* | *Ostertagia leptospicularis* | moderate confidence | ASV 165 |
| *Ostertagia* | *Ostertagia gruehneri* | Root | *Ostertagia* |  | ASV 191 |
| ***Spiculopteragia*** |  |  |  |  |  |
| *Spiculopteragia spiculoptera* | *Spiculopteragia spiculoptera* | *Spiculopteragia spiculoptera*  *(99.5%-99.5%-99.1%)* | *Spiculopteragia spiculoptera* | high confidence | ASV 184 |
| *Spiculopteragia spiculoptera* | *Spiculopteragia spiculoptera* | *Spiculopteragia*  *(99.3%-99.0%-97.9%)*  *(99.0%-98.6%-98.3%)*  *(98.6%-98.3%-97.9%)*  *(98.6%-98.3%-97.9%)*  *(98.6%-98.3%-97.9%)*  *(99.0%-98.6%-97.6%)*  *(99.0%-98.6%-97.6%)*  *(98.6%-98.3%-97.9%)*  *(98.6%-98.3%-97.9%)*  *(98.6%-98.3%-97.9%)*  *(98.6%-98.3%-97.9%)*  *(98.6%-98.3%-97.9%)*  *(98.6%-98.3%-97.9%)*  *(98.6%-98.3%-97.9%)* | *Spiculopteragia spiculoptera* | moderate confidence | ASV 5 ASV 6  ASV 38 ASV 43 ASV 66 ASV 90 ASV 95  ASV 105 ASV 109 ASV 114 ASV 127 ASV 130 ASV 132 ASV 222 |
| *Spiculopteragia* | *Spiculopteragia spiculoptera* | Root | *Spiculopteragia* |  | ASV 172 |
| ***Teladorsagia*** |  |  |  |  |  |
| *Teladorsagia circumcincta* | *Teladorsagia circumcincta* | *Teladorsagia circumcincta*  *(99.7%-99.0%-99.3%)*  *100%-100%-100%)*  *(99.7%-99.7%-99.7%)* | *Teladorsagia circumcincta* | high confidence | ASV 25 ASV 29 ASV 141 |
| ***Trichostrongylus*** |  |  |  |  |  |
| *Trichostrongylus axei* | *Trichostrongylus axei* | *Trichostrongylus axei*  *(100%-100%-100%)*  *(99.3%-99.3%-99.3%)*  *(98.6%-98.6%-98.6%)*  *(99.0%-99.0%-99.5%)*  *99.7%-99.7%-99.7%)*  *99.7%-99.7%-99.7%)*  *99.7%-99.7%-99.7%)* | *Trichostrongylus axei* | high confidence | ASV 4  ASV 16 ASV 64  ASV 78 ASV 79 ASV 80 ASV 136 |
| *Trichostrongylus colubriformis* | *Trichostrongylus colubriformis* | *Trichostrongylus colubriformis*  *(100%-100%-100%)* | *Trichostrongylus colubriformis* | high confidence | ASV 17 |
| *Trichostrongylus* | *Trichostrongylus vitrinus* | *Trichostrongylus*  *(97.2%-96.9%-96.2%)* | *Trichostrongylus* |  | ASV 202 |
| *Trichostrongylus* | *Trichostrongylus* | *Trichostrongylus vitrinus*  *(98.7%-94.6%-98.7%)* | *Trichostrongylus* |  | ASV 149 |
| *Trichostrongylus* | *Trichostrongylus* | *Trichostrongylus*  *(98.6%-98.3%-97.6%)*  *(97.6%-97.2%-96.5%)*  *(96.2%-96.2%-95.8%)*  *(96.9%-96.2%-95.8%)*  *(96.5%-95.8%-95.5%)*  *(97.2%-96.9%-96.2%)*  *(97.2%-96.9%-96.5%)*  *(96.9%-96.5%-95.8%)*  *(97.2%-96.9%-96.2%)*  *(97.2%-96.9%-96.2%)*  *(97.6%-97.2%-97.5%)*  *(97.6%-97.2%-96.9%)*  *(97.4%-97.0%-97.0%)*  *(97.2%-96.9%-96.2%)* | *Trichostrongylus* |  | ASV 2 ASV 3  ASV 11  ASV 13 ASV 18  ASV 33 ASV 57 ASV 84 ASV 92  ASV 102 ASV 135 ASV 169 ASV 177 ASV 180 |
| ***Varestrongylus*** |  |  |  |  |  |
| *Varestrongylus capreoli* | *Varestrongylus capreoli* | *Varestrongylus capreoli*  *(99.1%-100%-99.1%)*  *(100%-100%-98.8%)*  *(99.8%-99.8%-98.6%)*  *(98.6%-99.5%-98.6%)*  *(99.5%-99.5%-98.4%)*  *(99.8%-99.8%-98.6%)* | *Varestrongylus capreoli* | high confidence | ASV 41 ASV 44 ASV 62  ASV 143 ASV 148 ASV 156 |
| Root | *Varestrongylus capreoli* | *Varestrongylus capreoli*  *100%-90.8%-89.6%)* | *Varestrongylus capreoli* | moderate confidence | ASV 183 |
| *Varestrongylus capreoli* | *Varestrongylus capreoli* | *Varestrongylus capreoli*  *(93.4%-94.1%-93.2%)*  *(95.1%-93.7%-93.7%)*  *(93.6%-93.9%-93.4%)* | *Varestrongylus* |  | ASV 101  ASV 124  ASV 234 |
| Root | *Varestrongylus capreoli* | *Varestrongylus capreoli*  *(95.7%-91.1%-89.8%)* | *Varestrongylus* |  | ASV 147 |
| ***Strongylida*** |  |  |  |  |  |
| Root | Strongylida | Root | Root |  | ASV 146 ASV 152 ASV 201 |

*Assignment verified by a second search with BLASTn directly online (07/09/2021)

**Figure S2.** Read relative frequencies of GIN at taxa level in samples of 19 recaptured wild roe deer (*Capreolus capreolus)* from two isolated populations. Each taxon is defined by one color. The data are split based on site location (Chizé or Trois Fontaines) and individuals captured in 2018 and 2019.


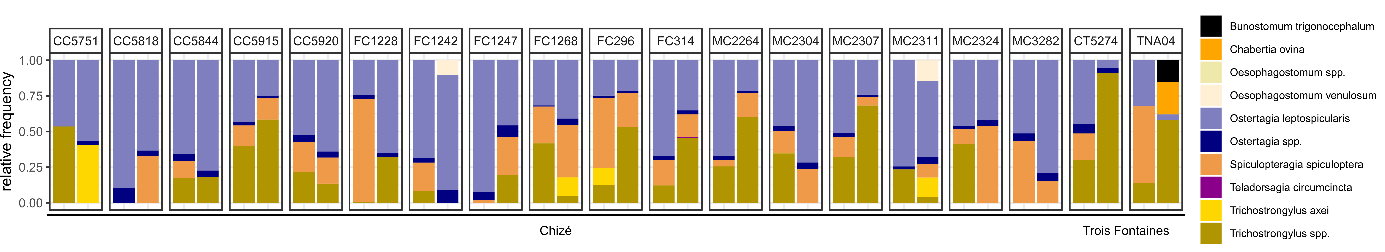


**Table S2a.** Results of Kruskal-Wallis tests testing the relationship between alpha diversity (estimated with richness, Simpson (1-D) and Shannon indices) computed for 2 taxonomic resolutions (Taxa, ASV), and year of sampling (2018 or 2019).

| **Taxonomic resolution** | **Diversity index** | **Χ²** | **P** |
| --- | --- | --- | --- |
| **ASVs** | Richness | 3.20 | - |
|  | Simpson | 4.08 | * |
|  | Shannon | 4.41 | * |
| **Taxa** | Richness | 0.92 | - |
|  | Simpson | 2.03 | - |
|  | Shannon | 1.23 | - |

**Table S2b.** Results of Spearman's correlation testing the relationship between alpha diversity (estimated with richness, Simpson (1-D) and Shannon indices) computed for 2 taxonomic resolutions (Taxa, ASV), and the number of nematode larvae L3.

| **Taxonomic resolution** | **Diversity index** | **ρ** | **P** |
| --- | --- | --- | --- |
| **ASVs** | Richness | 0.09 | - |
|  | Simpson | 0.09 | - |
|  | Shannon | 0.10 | - |
| **Taxa** | Richness | 0.09 | - |
|  | Simpson | 0.11 | - |
|  | Shannon | 0.10 | - |

**Table S2c.** Results of Spearman's testing the relationship between alpha diversity (estimated with richness, Simpson (1-D) and Shannon indices) computed for 2 taxonomic resolutions (Taxa, ASV), and the number of nematode eggs per gram.

| **Taxonomic resolution** | **Diversity index** | **ρ** | **p** |
| --- | --- | --- | --- |
| **ASVs** | Richness | -0.04 | - |
|  | Simpson | 0.06 | - |
|  | Shannon | 0.03 | - |
| **Taxa** | Richness | -0.08 | - |
|  | Simpson | 0.04 | - |
|  | Shannon | 0.01 | - |

**Table S2d.** Results of PerMANOVA testing the relationship between Bray Curtis dissimilarity computed for 2 taxonomic resolutions (Taxa, ASV) and the year, number of L3 or number of eggs per gram.

| **Taxonomic resolution** | **models** | **Variables** | **R²** | **F-value** | **P** |
| --- | --- | --- | --- | --- | --- |
| **ASVs** | $\beta\sim year$ | residuals | 0.99 | - | - |
|  |  | year | 0.01 | 1.21 | - |
|  | $\beta\sim epg$ | residuals | 1.00 | - | - |
|  |  | epg | 0.00 | 0.55 | - |
|  | $\beta\sim nb. of L3$ | residuals | 0.99 | - | - |
|  |  | nb. of L3 | 0.01 | 0.75 | - |
| **Taxa** | $\beta\sim year$ | residuals | 0.99 | - | - |
|  |  | year | 0.01 | 1.25 | - |
|  | $\beta\sim epg$ | residuals | 1.00 | - | - |
|  |  | epg | 0.00 | 0.60 | - |
|  | $\beta\sim nb. of L3$ | residuals | 1.00 | - | - |
|  |  | nb. of L3 | 0.00 | 0.40 | - |

**Figure S3.** Unrooted maximum likelihood tree of ASVs observed in wild roe deer (*Capreolus capreolus)* from two isolated populations. Branch lengths are modified with square root correction. Each color corresponds to a different genus.

**
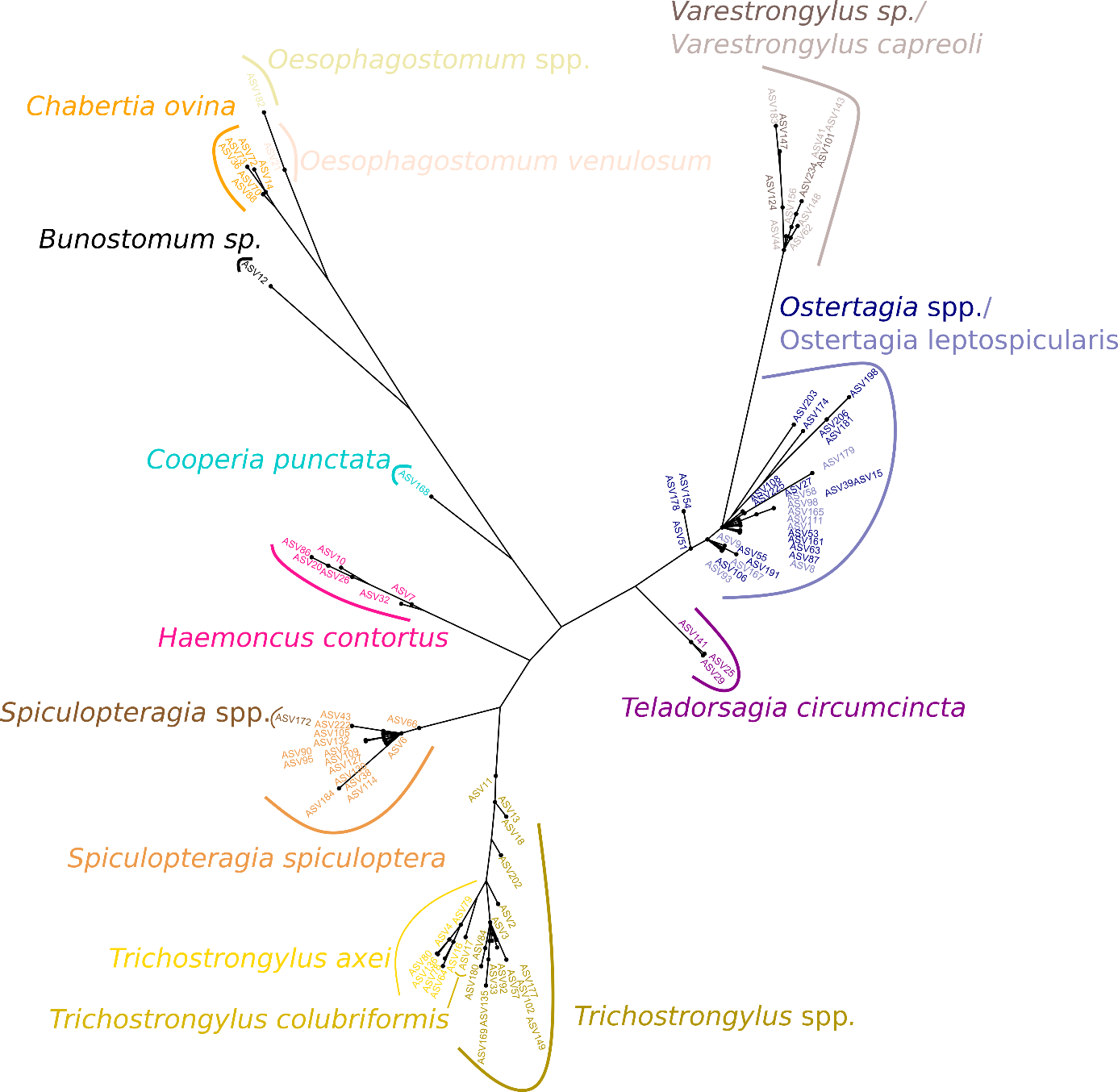
**

**Figure S4.** Sample accumulation curves for ASV and Taxa of the total data set (without the lungworm *Varestrongylus*).


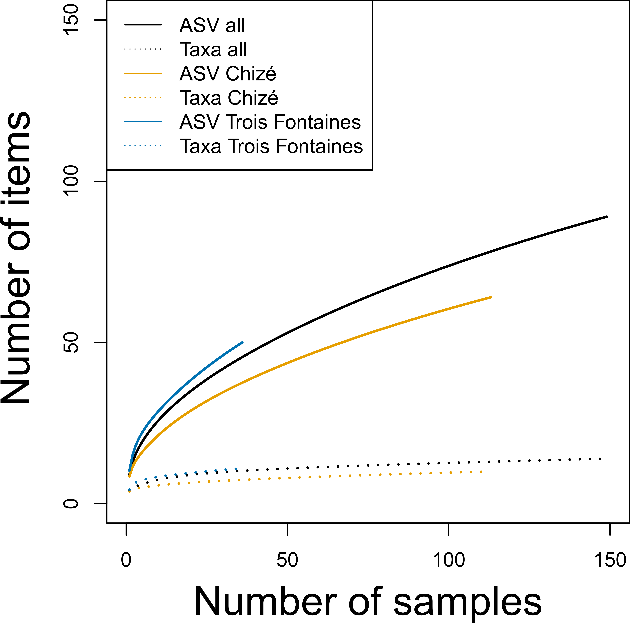


**Table S3a.** Set of generalized linear models sorted by AICc value. Alpha diversity, based on the total richness of GIN, was modeled with all possible combinations of age, sex and site variables and their interactions. The best model is highlighted in bold (i.e., the most parsimonious model among those with ΔAICc≤2).

| Taxonomic resolution | Generalized linear models | df | AICc | ΔAICc | weight |
| --- | --- | --- | --- | --- | --- |
| ASVs | $\boldsymbol{\alpha\sim site}$ | **2** | **632.93** | **0** | **0.17** |
|  | $\alpha\sim age+site+age\times site$ | 4 | 633.26 | 0.33 | 0.15 |
|  | $\alpha\sim age+sex+site+age\times site$ | 5 | 633.69 | 0.76 | 0.12 |
|  | $\alpha\sim sex+site$ | 3 | 633.74 | 0.81 | 0.12 |
|  | $\alpha\sim age+sex+site+age\times site+sex\times site$ | 6 | 634.24 | 1.31 | 0.09 |
|  | $\alpha\sim sex+site+sex\times site$ | 4 | 634.24 | 1.31 | 0.09 |
|  | $\alpha\sim age+site$ | 3 | 634.91 | 1.98 | 0.06 |
|  | $\alpha\sim age+sex+site+age\times sex+age\times site$ | 6 | 635.32 | 2.39 | 0.05 |
|  | $\alpha\sim age+sex+site$ | 4 | 635.81 | 2.88 | 0.04 |
|  | $\alpha\sim age+sex+site+sex\times site$ | 5 | 636.27 | 3.34 | 0.03 |
|  | $\alpha\sim age+sex+site+age\times site+sex\times site+sex\times age$ | 7 | 636.33 | 3.40 | 0.03 |
|  | $\alpha\sim age+sex+site+age\times sex$ | 5 | 637.25 | 4.32 | 0.02 |
|  | $\alpha\sim age+sex+site+age\times sex+sex\times site$ | 6 | 638.17 | 5.24 | 0.01 |
|  | $\alpha\sim sex$ | 2 | 640.11 | 7.18 | 0 |
|  | $\alpha\sim1$ | 1 | 640.22 | 7.29 | 0 |
|  | $\alpha\sim age$ | 2 | 641.30 | 8.37 | 0 |
|  | $\alpha\sim age+sex$ | 3 | 641.52 | 8.59 | 0 |
|  | $\alpha\sim age+sex+age\times sex$ | 4 | 643.28 | 10.35 | 0 |
| Taxa | $\alpha\sim site$ | 2 | 459.06 | 0 | 0.22 |
|  | $\boldsymbol{\alpha\sim1}$ | **1** | **459.22** | **0.16** | **0.20** |
|  | $\alpha\sim sex$ | 2 | 460.64 | 1.58 | 0.10 |
|  | $\alpha\sim sex+site$ | 3 | 460.75 | 1.69 | 0.09 |
|  | $\alpha\sim age$ | 2 | 461.12 | 2.06 | 0.08 |
|  | $\alpha\sim age+site$ | 3 | 461.15 | 2.09 | 0.08 |
|  | $\alpha\sim sex+site+sex\times site$ | 4 | 461.73 | 2.67 | 0.06 |
|  | $\alpha\sim age+sex$ | 3 | 462.63 | 3.58 | 0.04 |
|  | $\alpha\sim age+sex+site$ | 4 | 462.88 | 3.82 | 0.03 |
|  | $\alpha\sim age+site+age\times site$ | 4 | 463.16 | 4.10 | 0.03 |
|  | $\alpha\sim age+sex+site+sex\times site$ | 5 | 463.88 | 4.82 | 0.02 |
|  | $\alpha\sim age+sex+age\times sex$ | 4 | 464.07 | 5.01 | 0.02 |
|  | $\alpha\sim age+sex+site+age\times sex$ | 5 | 464.13 | 5.07 | 0.02 |
|  | $\alpha\sim age+sex+site+age\times site$ | 5 | 464.88 | 5.82 | 0.01 |
|  | $\alpha\sim age+sex+site+age\times sex+sex\times site$ | 6 | 465.57 | 6.51 | 0.01 |
|  | $\alpha\sim age+sex+site+age\times site+sex\times site$ | 6 | 465.91 | 6.85 | 0.01 |
|  | $\alpha\sim age+sex+site+age\times sex+age\times site$ | 6 | 466.2 | 7.14 | 0.01 |
|  | $\alpha\sim age+sex+site+ age\times sex+age\times site+sexe\times site$ | 7 | 467.69 | 8.63 | 0 |

**Table S3b.** Set of generalized linear models sorted by AICc value. Alpha diversity, based on the Shannon index, was modeled with all possible combinations of age, sex and site variables and their interactions. The best model is highlighted in bold (i.e., the most parsimonious model among those with ΔAICc≤2).

| Taxonomic resolution | Generalized linear models | df | AICc | ΔAICc | weight |
| --- | --- | --- | --- | --- | --- |
| ASVs | $\alpha\sim age+sex+site+age\times site+sex\times site$ | 7 | 78.41 | 0 | 0.24 |
|  | $\alpha\sim age+sex+site+age\times site+sex\times site+sex\times age$ | 8 | 78.75 | 0.34 | 0.20 |
|  | $\boldsymbol{\alpha\sim age+site+age\times site}$ | **5** | **78.94** | **0.53** | **0.18** |
|  | $\alpha\sim age+sex+site+age\times sex+age\times site$ | 7 | 79.92 | 1.51 | 0.11 |
|  | $\alpha\sim age+sex+site+age\times site$ | 6 | 81.10 | 2.69 | 0.06 |
|  | $\alpha\sim site$ | 3 | 82.03 | 3.62 | 0.04 |
|  | $\alpha\sim sex+site+ sex\times site$ | 5 | 82.21 | 3.80 | 0.04 |
|  | $\alpha\sim age+sex+site+sex\times site$ | 6 | 82.28 | 3.87 | 0.03 |
|  | $\alpha\sim age+sex+site+age\times sex+sex\times site$ | 7 | 82.36 | 3.95 | 0.03 |
|  | $\alpha\sim age+site$ | 4 | 82.62 | 4.21 | 0.03 |
|  | $\alpha\sim age+sex+site+age\times sex$ | 6 | 83.33 | 4.91 | 0.02 |
|  | $\alpha\sim sex+site$ | 4 | 84.16 | 5.74 | 0.01 |
|  | $\alpha\sim age+sex+site$ | 5 | 84.78 | 6.37 | 0.01 |
|  | $\alpha\sim age$ | 3 | 91.57 | 13.16 | 0 |
|  | $\alpha\sim1$ | 2 | 93.17 | 14.75 | 0 |
|  | $\alpha\sim age+sex+age\times sex$ | 5 | 93.42 | 15 | 0 |
|  | $\alpha\sim age+sex$ | 4 | 93.64 | 15.23 | 0 |
|  | $\alpha\sim sex$ | 3 | 95.05 | 16.64 | 0 |
| Taxa | $\boldsymbol{\alpha\sim site}$ | **3** | **68.87** | **0** | **0.24** |
|  | $\alpha\sim sexe+site+sex\times site$ | 5 | 68.88 | 0 | 0.24 |
|  | $\alpha\sim age+sex+site+sex\times site$ | 6 | 70.87 | 2 | 0.09 |
|  | $\alpha\sim age+site$ | 4 | 70.95 | 2.07 | 0.08 |
|  | $\alpha\sim sex+site$ | 4 | 70.99 | 2.12 | 0.08 |
|  | $\alpha\sim1$ | 2 | 72.02 | 3.14 | 0.05 |
|  | $\alpha\sim age+sex+site+age\times site+sex\times site$ | 7 | 72.72 | 3.85 | 0.03 |
|  | $\alpha\sim age+site+age\times site$ | 5 | 72.73 | 3.85 | 0.03 |
|  | $\alpha\sim age+sex+site+age\times sex+sex\times site$ | 7 | 72.77 | 3.89 | 0.03 |
|  | $\alpha\sim age+sex+site$ | 5 | 73.09 | 4.22 | 0.03 |
|  | $\alpha\sim age$ | 3 | 73.61 | 4.73 | 0.02 |
|  | $\alpha\sim sex$ | 3 | 74.10 | 5.22 | 0.02 |
|  | $\alpha\sim age+sex+site+age\times sex$ | 6 | 74.20 | 5.32 | 0.02 |
|  | $\alpha\sim age+sex+site+age\times sex+age\times site+sex\times site$ | 8 | 74.69 | 5.81 | 0.01 |
|  | $\alpha\sim age+sex+site+age\times site$ | 6 | 74.92 | 6.04 | 0.01 |
|  | $\alpha\sim age+sex$ | 4 | 75.73 | 6.86 | 0.01 |
|  | $\alpha\sim age+sex+site+age\times sex+age\times site$ | 7 | 76.11 | 7.23 | 0.01 |
|  | $\alpha\sim age+sex+age\times sex$ | 5 | 77.19 | 8.32 | 0 |

**Table S3c.** Set of generalized linear models sorted by AICc value. Alpha diversity, based on the Simpson index ((1-D), was modeled with all possible combinations of age, sex and site variables and their interactions. The best model is highlighted in bold (i.e., the most parsimonious model among those with ΔAICc≤2).

| taxonomic resolution | Generalized linear models | df | AICc | ΔAICc | weight |
| --- | --- | --- | --- | --- | --- |
| ASVs | $\alpha\sim age+sex+site+age\times sex+age\times site$ | 7 | -213.6 | 0 | 0.13 |
|  | $\alpha\sim age+sex+site+age\times sex+age\times site+sex\times site$ | 8 | -213.16 | 0.44 | 0.10 |
|  | $\alpha\sim age+sex+site+age\times sex$ | 6 | -213.13 | 0.47 | 0.10 |
|  | $\alpha\sim age+site+age\times site$ | 5 | -213.03 | 0.57 | 0.10 |
|  | $\alpha\sim age+sex+site+age\times sex+sex\times site$ | 7 | -212.31 | 1.28 | 0.07 |
|  | $\boldsymbol{\alpha\sim site}$ | **3** | **-212.25** | **1.35** | **0.07** |
|  | $\alpha\sim age+sex+site+age\times site+sex\times site$ | 7 | -212.15 | 1.45 | 0.06 |
|  | $\alpha\sim age+sex+site+age\times site$ | 6 | -211.99 | 1.61 | 0.06 |
|  | $\alpha\sim age+site$ | 4 | -211.71 | 1.88 | 0.05 |
|  | $\alpha\sim age+sex+site+sex\times site$ | 6 | -211.49 | 2.11 | 0.04 |
|  | $\alpha\sim sex+site+sex\times site$ | 5 | -211.38 | 2.22 | 0.04 |
|  | $\alpha\sim age+sex+age\times sex$ | 5 | -211.30 | 2.30 | 0.04 |
|  | $\alpha\sim sex+site$ | 4 | -211.06 | 2.53 | 0.04 |
|  | $\alpha\sim age+sex+site$ | 5 | -210.92 | 2.67 | 0.03 |
|  | $\alpha\sim age$ | 3 | -210.79 | 2.81 | 0.03 |
|  | $\alpha\sim1$ | 2 | -209.80 | 3.80 | 0.02 |
|  | $\alpha\sim age+sex$ | 4 | -209.52 | 4.07 | 0.02 |
|  | $\alpha\sim sex$ | 3 | -208.09 | 5.50 | 0.01 |
| Taxa | $\alpha\sim site$ | 3 | -86.51 | 0 | 0.19 |
|  | $\boldsymbol{\alpha\sim1}$ | 2 | -86.27 | 0.25 | 0.17 |
|  | $\alpha\sim sex+site+sex\times site$ | 5 | -85.83 | 0.68 | 0.13 |
|  | $\alpha\sim sex+site$ | 4 | -84.81 | 1.70 | 0.08 |
|  | $\alpha\sim age$ | 3 | -84.51 | 2 | 0.07 |
|  | $\alpha\sim age+site$ | 4 | -84.45 | 2.06 | 0.07 |
|  | $\alpha\sim sex$ | 3 | -84.38 | 2.13 | 0.07 |
|  | $\alpha\sim age+sex+site+sex\times site$ | 6 | -83.89 | 2.63 | 0.05 |
|  | $\alpha\sim age+sex+site$ | 5 | -82.75 | 3.76 | 0.03 |
|  | $\alpha\sim age+sex$ | 4 | -82.67 | 3.84 | 0.03 |
|  | $\alpha\sim age+site+age\times site$ | 5 | -82.48 | 4.03 | 0.03 |
|  | $\alpha\sim age+sex+site+age\times sex+sex\times site$ | 7 | -82.03 | 4.49 | 0.02 |
|  | $\alpha\sim age+sex+site+age\times site+sex\times site$ | 7 | -81.80 | 4.71 | 0.02 |
|  | $\alpha\sim age+sex+site+age\times sex$ | 6 | -81.58 | 4.94 | 0.02 |
|  | $\alpha\sim age+sex+age\times sex$ | 5 | -81.27 | 5.25 | 0.01 |
|  | $\alpha\sim age+sex+site+age\times site$ | 6 | -80.69 | 5.82 | 0.01 |
|  | $\alpha\sim age+sex+site+age\times sex+age\times site+sex\times site$ | 8 | -79.89 | 6.63 | 0.01 |
|  | $\alpha\sim age+sex+site+age\times sex+age\times site$ | 7 | -79.45 | 7.07 | 0.01 |

**Table S3d.** Set of perMANOVA models sorted by AICc value. Bray Curtis dissimilarity is modeled with all possible combinations of age, sex and site variables and their interactions. The best model is highlighted in bold (i.e., the most parsimonious model among those with ΔAICc≤2).

| taxonomic resolution | Generalized linear models | k | AICc | ΔAICc | weight |
| --- | --- | --- | --- | --- | --- |
| ASVs | $\beta\sim age+site+age\times site$ | 4 | 372.6 | 0 | 0.21 |
|  | $\beta\sim age+sex+site+age\times sex+age\times site$ | 6 | 373.37 | 0.77 | 0.14 |
|  | $\beta\sim age+sex+site+age\times site$ | 5 | 373.82 | 1.22 | 0.12 |
|  | $\beta\sim age+sex+site+age\times site+sex\times site+sex\times age$ | 7 | 373.85 | 1.25 | 0.11 |
|  | $\beta\sim age+sex+site+age\times site+sex\times site$ | 6 | 374.06 | 1.46 | 0.10 |
|  | $\boldsymbol{\beta\sim age+site}$ | **3** | **374.39** | **1.79** | **0.09** |
|  | $\beta\sim age+sex+site+age\times sex$ | 5 | 374.81 | 2.21 | 0.07 |
|  | $\beta\sim age+sex+site+age\times sex+sex\times site$ | 6 | 375.32 | 2.72 | 0.05 |
|  | $\beta\sim age+sex+site$ | 4 | 375.34 | 2.74 | 0.05 |
|  | $\beta\sim age+sex+site+sex\times site$ | 5 | 375.6 | 3 | 0.05 |
|  | $\beta\sim site$ | 2 | 384.67 | 12.07 | 0 |
|  | $\beta\sim sex+site$ | 3 | 385.84 | 13.24 | 0 |
|  | $\beta\sim sex+site+ sex\times site$ | 4 | 386.47 | 13.87 | 0 |
|  | $\beta\sim age$ | 2 | 404.09 | 31.49 | 0 |
|  | $\beta\sim age+sex$ | 3 | 405 | 32.40 | 0 |
|  | $\beta\sim age+sex+age\times sex$ | 4 | 405.31 | 32.71 | 0 |
|  | $\beta\sim1$ | 1 | 412.36 | 39.76 | 0 |
|  | $\beta\sim sex$ | 2 | 413.39 | 40.79 | 0 |
| Taxa | $\beta\sim age+sex+site+age\times sex+age\times site$ | 6 | 344.26 | 0 | 0.19 |
|  | $\beta\sim age+sex+site+age\times site+sex\times site+sex\times age$ | 7 | 344.68 | 0.42 | 0.15 |
|  | $\beta\sim age+sex+site+age\times sex$ | 5 | 345 | 0.74 | 0.13 |
|  | $\boldsymbol{\beta\sim age+site+age\times site}$ | **4** | **345.34** | **1.08** | **0.11** |
|  | $\beta\sim age+sex+site+age\times sex+sex\times site$ | 6 | 345.41 | 1.15 | 0.11 |
|  | $\beta\sim age+sex+site+age\times site$ | 5 | 346.07 | 1.81 | 0.08 |
|  | $\beta\sim age+site$ | 3 | 346.28 | 2.02 | 0.07 |
|  | $\beta\sim age+sex+site+age\times site+sex\times site$ | 6 | 346.63 | 2.37 | 0.06 |
|  | $\beta\sim age+sex+site$ | 4 | 346.77 | 2.51 | 0.05 |
|  | $\beta\sim age+sex+site+sex\times site$ | 5 | 347.33 | 3.07 | 0.04 |
|  | $\beta\sim site$ | 2 | 354.56 | 10.30 | 0 |
|  | $\beta\sim sex+site$ | 3 | 355.49 | 11.23 | 0 |
|  | $\beta\sim sex+site+ sex\times site$ | 4 | 355.96 | 11.70 | 0 |
|  | $\beta\sim age$ | 2 | 365.21 | 20.95 | 0 |
|  | $\beta\sim age+sex+age\times sex$ | 4 | 365.41 | 21.15 | 0 |
|  | $\beta\sim age+sex$ | 3 | 365.79 | 21.53 | 0 |
|  | $\beta\sim1$ | 1 | 376.51 | 32.25 | 0 |
|  | $\beta\sim sex$ | 2 | 377.28 | 33.02 | 0 |

**Table S4a.** Best Generalized linear models selected for taxa and diversity index (Richness, Simpson (1-D), Shannon). Gaussian families were used for Simpson and Shannon regression and Poisson family for Richness. The effect of site (Chizé as referent), age (adult as referent) and their interaction are reported when including in the selected model. Parameter estimate with SD are reported with the corresponding z-value (Poisson family) or t-value (Gaussian family) and p-value. Statistical significance is represented by * for P<0.05 and *** for P<0.001.

| **Taxonomic resolution** | **Diversity index** | **Best Model selected** | **Variables** | **Parameter estimate ± SE** | **z-val. or**  **t-val.** | **P** |
| --- | --- | --- | --- | --- | --- | --- |
| **Taxa** | Richness | $\alpha\sim1$ | intercept | 1.38 ± 0.04 | 31.32 | *** |
|  | Simpson | $\alpha\sim1$ | intercept | 0.05 ± 0.06 | 0.89 | - |
|  | Shannon | $\alpha\sim site$ | intercept | 0.88 ± 0.03 | 27.94 | *** |
|  |  |  | siteTF | 0.14 ± 0.06 | 2.29 | * |

**Table S4b.** Best perMANOVA models selected for taxa. Bray-Curtis dissimilarities matrix were used for regressions. The effect of site (Chizé as referent), age (adult as referent) and the interaction between them are reported when including in the selected model. R² are reported with the corresponding F-value and p-value. Statistical significance is represented by* for P<0.05 and *** for P<0.001.

| **Taxonomic resolution** | **Best Model selected** | **Variables** | **R²** | **F** | **P** |
| --- | --- | --- | --- | --- | --- |
| **Taxa** | $\beta\sim age+site+age\times site$ | residuals | 0.75 | - | - |
|  |  | site | 0.17 | 28.36 | *** |
|  |  | age | 0.06 | 10.71 | *** |
|  |  | age:site | 0.02 | 3.01 | * |

**Figure S5.** Non-metric Multidimensional Scaling (Taxa) of nemabiome based on 14 Taxa from the 130 roe deer of different age and sex sampled in Chizé and Trois Fontaines. The stress has a value of 0.10.

**
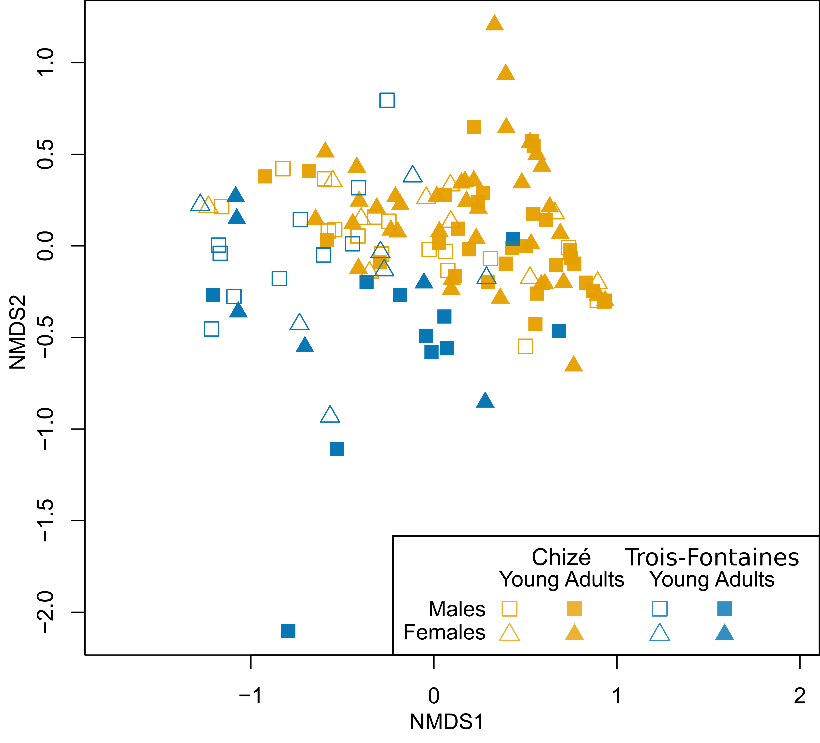
**

**Figure S6.** Heatmap of ASVs observed in wild roe deer (*Capreolus capreolus)* from two isolated populations. Each column corresponds to one sample of roe deer. Each row corresponds to an ASV with their assignment (B.t=*Bunostomum trigonocephalum*; C.o= *Chabertia ovina*; C.p=*Cooperia punctata*; H.c= *Haemonchus contortus*; Oe.v=*Oesophagostomum venulosum*; Os.l=*Ostertagia leptospicularis*; S.s=*Spiculopteragia spiculoptera*; T.ci=*Teladorsagia circumcincta*: T.a=*Trichostrongylus axei*; T.co=*Trichostrongylus colubriformis*). ASV names have the same color when they belong to the same genus. The data were split based on site location (Chizé or Trois Fontaines), age (young or adult roe deer) and sex (F: female and M: male). For each sample, the color indicates the read relative frequency of each ASV. Empty cells (white color) correspond to 0 reads at the end of the data curation.

**
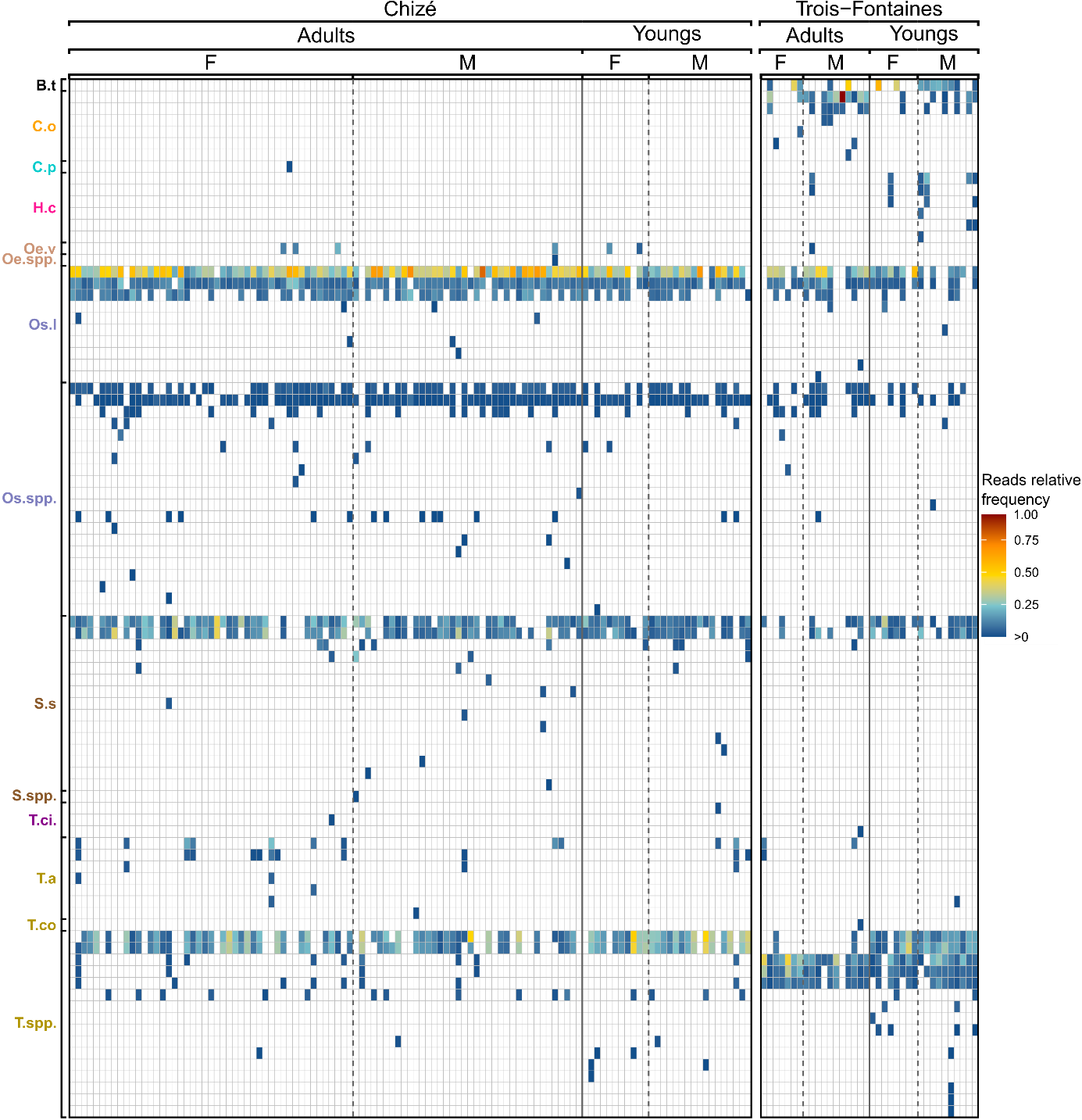
**
