# Supplementary material for: Metabarcoding in two isolated populations of wild roe deer (Capreolus capreolus) reveals variation in gastrointestinal nematode community composition between regions and among age classes
Source: Parasit Vectors. 2021 Dec 4;14:594. doi: 10.1186/s13071-021-05087-5 (PMC8642965; doi:10.1186/s13071-021-05087-5)
Supplement: Supplementary file 2 — Additional file 2. OTUs analysis. [file 13071_2021_5087_MOESM2_ESM.docx]

**Metabarcoding in two isolated populations of wild roe deer (*Capreolus capreolus)* reveals variation in gastrointestinal nematode community composition between regions and among age classes**

Camille Beaumelle^1,3^* and Libby Redman^2,^*, Jill de Rijke^2^, Janneke Wit^2^, Slimania Benabed^1,4^, François Debias^1^, Jeanne Duhayer^1^, Sylvia Pardonnet^1^, Marie-Thérèse Poirel^1,4^, Gilles Capron^5^, Stéphane Chabot^5^, Benjamin Rey^1^, Glenn Yannic^3^, John S Gilleard^2,^** and Gilles Bourgoin^1,4,^**

^1^Université de Lyon, Université Lyon 1, CNRS, Laboratoire de Biométrie et Biologie Evolutive UMR 5558, F-69100 Villeurbanne, France

^2^Comparative Biology and Experimental medicine, Host-Parasites Interactions Program, Faculty of Veterinary Medicine, University of Calgary, Calgary, Alberta, Canada.

^3^Université Grenoble Alpes, Université Savoie Mont Blanc, CNRS, LECA, 38000, Grenoble, France

^4^Université de Lyon, VetAgro Sup, Campus Vétérinaire de Lyon, F-69280 Marcy l’Etoile, France

^5^Office Français de la Biodiversité, F-75008 Paris, France

*These authors contributed equally to this work

**Co-senior authors

Correspondence: Beaumelle.camille@gmail.com

**A10 OTUs Analysis**

**Table a.**

Summary of OTUs observed in wild roe deer (*Capreolus capreolus)* from the isolated populations of Chizé and Trois Fontaines sampled in 2018 and 2019.

|  |  | 2018 | | 2019 | | Total  (N=149) |
| --- | --- | --- | --- | --- | --- | --- |
|  |  | **Chizé**  **(N=57)** | **Trois-Fontaines**  **(N=22)** | **Chizé**  **(N=56)** | **Trois-Fontaines**  **(N=14)** |  |
| OTUs | median [min-max] | 4 [1-8] | 6 [3-10] | 4 [1-8] | 5 [1-12] | 4 [1-12] |
|  | Total | 21 | 18 | 21 | 16 | 37 |

**Table b.** Set of generalized linear models for OTUs sorted by AICc value. Alpha diversity, based on the total GIN richness, Shannon and Simpson index was modeled with all possible combinations of age, sex and site variables and their interactions. The best model is highlighted in bold (i.e., the most parsimonious model among those with ΔAICc≤2).

| Taxonomic resolution | Generalized linear models | df | AICc | ΔAICc | weight |
| --- | --- | --- | --- | --- | --- |
| Richness | $\boldsymbol{\alpha\sim age+site+age\times site}$ | **4** | **485.51** | **0** | **0.33** |
|  | $\alpha\sim age+sex+site+age\times site$ | 5 | 487.43 | 1.92 | 0.13 |
|  | $\alpha\sim age+sex+site+age\times site+sex\times site$ | 6 | 487.64 | 2.13 | 0.12 |
|  | $\alpha\sim age+site$ | 3 | 487.72 | 2.21 | 0.11 |
|  | $\alpha\sim age+sex+site+age\times sex+age\times site$ | 6 | 488.47 | 2.96 | 0.08 |
|  | $\alpha\sim age+sex+site+age\times site+sex\times site+sex\times age$ | 7 | 489.46 | 3.95 | 0.05 |
|  | $\alpha\sim age+sex+site$ | 4 | 489.78 | 4.27 | 0.04 |
|  | $\alpha\sim age+sex+site+sex\times site$ | 5 | 489.85 | 4.34 | 0.04 |
|  | $\alpha\sim site$ | 2 | 489.88 | 4.37 | 0.04 |
|  | $\alpha\sim age+sex+site+age\times sex$ | 5 | 490.5 | 4.99 | 0.03 |
|  | $\alpha\sim age+sex+site+age\times sex+sex\times site$ | 6 | 491.3 | 5.79 | 0.02 |
|  | $\alpha\sim sex+site$ | 3 | 491.79 | 6.28 | 0.01 |
|  | $\alpha\sim sex+site+sex \times site$ | 4 | 492.39 | 6.87 | 0.01 |
|  | $\alpha\sim age$ | 2 | 495.65 | 10.14 | 0 |
|  | $\alpha\sim sex+age$ | 3 | 497.46 | 11.95 | 0 |
|  | $\alpha\sim age+sex+age\times sex$ | 4 | 498.75 | 13.24 | 0 |
|  | $\alpha\sim1$ | 1 | 501.57 | 16.06 | 0 |
|  | $\alpha\sim sex$ | 2 | 502.9 | 17.39 | 0 |
| Shannon | $\alpha\sim age+sex+site+ age\times sex+age\times site+sexe\times site$ | 8 | 123.24 | 0 | 0.31 |
|  | $\alpha\sim age+sex+site+age\times site+sex\times site$ | 7 | 123.46 | 0.23 | 0.27 |
|  | $\boldsymbol{\alpha\sim age+site+age\times site}$ | **5** | **125.19** | **1.95** | **0.12** |
|  | $\alpha\sim age+sex+site+age\times sex+age\times site$ | 7 | 125.19 | 1.96 | 0.11 |
|  | $\alpha\sim age+sex+site+age\times sex+sex\times site$ | 7 | 126.61 | 3.37 | 0.06 |
|  | $\alpha\sim age+sex+site+sex\times site$ | 6 | 127.11 | 3.87 | 0.04 |
|  | $\alpha\sim age+sex+site+age\times site$ | 6 | 127.29 | 4.05 | 0.04 |
|  | $\alpha\sim age+sex+site+age\times sex$ | 6 | 128.33 | 5.09 | 0.02 |
|  | $\alpha\sim age+site$ | 4 | 128.84 | 5.61 | 0.02 |
|  | $\alpha\sim age+sex+site$ | 5 | 130.71 | 7.47 | 0.01 |
|  | $\alpha\sim age$ | 3 | 136.15 | 12.91 | 0 |
|  | $\alpha\sim age+sex+age\times sex$ | 5 | 137.17 | 13.94 | 0 |
|  | $\alpha\sim age+sex$ | 4 | 138.21 | 14.98 | 0 |
|  | $\alpha\sim site$ | 3 | 140.92 | 17.68 | 0 |
|  | $\alpha\sim sex+site+sex\times site$ | 5 | 141.54 | 18.3 | 0 |
|  | $\alpha\sim sex+site$ | 4 | 143.02 | 19.78 | 0 |
|  | $\alpha\sim1$ | 2 | 152.76 | 29.53 | 0 |
|  | $\alpha\sim sex$ | 3 | 154.8 | 31.56 | 0 |
| Simpson | $\alpha\sim age+sex+site+sex\times site$ | 6 | -50.4 | 0 | 0.14 |
|  | $\alpha\sim age+sex+site+age\times site+sex\times site$ | 7 | -50.11 | 0.29 | 0.12 |
|  | $\alpha\sim age+sex+site+age\times sex+sex\times site$ | 7 | -49.97 | 0.44 | 0.11 |
|  | $\alpha\sim age+sex+site+ age\times sex+age\times site+sexe\times site$ | 8 | -49.8 | 0.6 | 0.1 |
|  | $\alpha\sim age+site+age\times site$ | 5 | -49.64 | 0.76 | 0.09 |
|  | $\alpha\sim age+site$ | 4 | -49.61 | 0.79 | 0.09 |
|  | $\alpha\sim age+sex+site+age\times sex$ | 6 | -49.32 | 1.08 | 0.08 |
|  | $\alpha\sim age+sex+site+age\times sex+age\times site$ | 7 | -48.79 | 1.62 | 0.06 |
|  | $\alpha\sim age+sex+site$ | 5 | -48.52 | 1.88 | 0.05 |
|  | $\alpha\sim age+sex+site+age\times site$ | 6 | -48.32 | 2.08 | 0.05 |
|  | $\alpha\sim age$ | 3 | -48.14 | 2.26 | 0.04 |
|  | $\alpha\sim age+sex+age\times sex$ | 5 | -46.92 | 3.48 | 0.02 |
|  | $\alpha\sim age+sex$ | 4 | -46.65 | 3.75 | 0.02 |
|  | $\alpha\sim site$ | 3 | -40.79 | 9.62 | 0 |
|  | $\alpha\sim sex+site+sex\times site$ | 5 | -39.53 | 10.88 | 0 |
|  | $\alpha\sim sex+site$ | 4 | -39.05 | 11.36 | 0 |
|  | $\alpha\sim1$ | 2 | -35.98 | 14.43 | 0 |
|  | $\alpha\sim sex$ | 3 | -33.95 | 16.45 | 0 |

**Table c.** The best generalized linear models selected for OTUs and diversity index (Richness, Simpson (1-D), Shannon). Gaussian families were used for Simpson and Shannon regression and Poisson family for Richness. The effect of site (Chizé as referent), age (adult as referent) and their interaction are reported when including in the selected model. Parameter estimate with SD are reported with the corresponding z-value (Poisson family) or t-value (Gaussian family) and p-value. Statistical significance is represented by * for P<0.05, **for P<0.01 and *** for P<0.001.

| **Taxonomic resolution** | **Diversity index** | **Best Model selected** | **Variables** | **Parameter estimate ± SE** | **z-val. or**  **t-val.** | **P** |
| --- | --- | --- | --- | --- | --- | --- |
| **OTUs** | Richness | $\alpha\sim age+site+age\times site$ | intercept | 1.32 ± 0.06 | 21.26 | *** |
|  |  |  | siteTF | 0.11±0.13 | 0.85 | - |
|  |  |  | ageY | 0.04±0.12 | 0.35 | - |
|  |  |  | ageY:siteTF | 0.40±0.19 | 2.06 | * |
|  | Simpson | $\alpha\sim age+site$ | intercept | -0.06 ± 0.05 | 1.13 | * |
|  |  |  | siteTF | 0.32 ± 0.17 | 1.86 | - |
|  |  |  | ageY | 0.52 ± 0.16 | 3.27 | ** |
|  | Shannon | $\alpha\sim age+site+age\times site$ | intercept | 0.91 ± 0.05 | 19.88 | *** |
|  |  |  | siteTF | 0.08 ± 0.10 | 0.81 | - |
|  |  |  | ageY | 0.17 ± 0.09 | 1.90 | - |
|  |  |  | ageY:siteTF | 0.38 ± 0.16 | 2.40 | * |

**Table d.** Set of perMANOVA models for OTUs sorted by AICc value. Bray Curtis dissimilarity is modeled with all possible combinations of age, sex, and site variables and interactions. The best model is highlighted in bold (i.e., the most parsimonious model among those with ΔAICc≤2).

| taxonomic resolution | Generalized linear models | k | AICc | ΔAICc | weight |
| --- | --- | --- | --- | --- | --- |
| OTUs | $\boldsymbol{\beta\sim age+site+age\times site}$ | **4** | **353.08** | **0** | **0.21** |
|  | $\beta\sim age+sex+site+age\times sex+age\times site$ | 6 | 353.51 | 0.43 | 0.17 |
|  | $\beta\sim age+sex+site+age\times site+sex\times site+sex\times age$ | 7 | 353.9 | 0.82 | 0.14 |
|  | $\beta\sim age+sex+site+age\times site$ | 5 | 354.22 | 1.14 | 0.12 |
|  | $\beta\sim age+sex+site+age\times site+sex\times site$ | 6 | 354.44 | 1.36 | 0.11 |
|  | $\beta\sim age+site$ | 3 | 355.35 | 2.27 | 0.07 |
|  | $\beta\sim age+sex+site+age\times sex$ | 5 | 355.43 | 2.35 | 0.06 |
|  | $\beta\sim age+sex+site+age\times sex+sex\times site$ | 6 | 355.85 | 2.77 | 0.05 |
|  | $\beta\sim age+sex+site$ | 4 | 356.2 | 3.12 | 0.04 |
|  | $\beta\sim age+sex+site+sex\times site$ | 5 | 356.45 | 3.37 | 0.04 |
|  | $\beta\sim site$ | 2 | 367.34 | 14.26 | 0 |
|  | $\beta\sim sex+site$ | 3 | 368.49 | 15.41 | 0 |
|  | $\beta\sim sex+site+ sex\times site$ | 4 | 369.13 | 16.05 | 0 |
|  | $\beta\sim age$ | 2 | 389.34 | 36.26 | 0 |
|  | $\beta\sim age+sex$ | 3 | 390.21 | 37.13 | 0 |
|  | $\beta\sim age+sex+age\times sex$ | 4 | 390.47 | 37.39 | 0 |
|  | $\beta\sim1$ | 1 | 398.87 | 45.79 | 0 |
|  | $\beta\sim sex$ | 2 | 399.89 | 46.81 | 0 |

**Table e.** Best perMANOVA models selected for OTUs. Bray-Curtis dissimilarities matrix were used for regressions. The effect of site (Chizé as referent), age (adult as referent) and their interactions are reported when included in the selected model. R² are reported with the corresponding F-value and p-value. Statistical significance is represented by **for P<0.01 and *** for P<0.001.

| **Taxonomic resolution** | **Best Model selected** | **Variables** | **R²** | **F** | **P** |
| --- | --- | --- | --- | --- | --- |
| **OTUs** | $\alpha\sim age+site+age\times site$ | residuals | 0.67 | - | - |
|  |  | site | 0.23 | 42.83 | *** |
|  |  | age | 0.08 | 14.92 | *** |
|  |  | age:site | 0.02 | 4.34 | ** |
